# Why pharmacogenomic biomarkers for chemotherapy-induced peripheral neuropathy fail: a systematic review of genetic associations, replication, and clinical translation

**DOI:** 10.3389/fphar.2026.1842379

**Published:** 2026-07-09

**Authors:** Aditya Maganti, Ishara Rankothge, Hunter Sakadales, Ethan Kok, James Britton, Neil Rao, Svetlana Shatunova, Katherine Shimell, Paula Manuela Rojas Zambrano, Susanna B. Park, Daniel Schweitzer, E-Liisa Laakso, Loic Yengo, Irina Vetter, Hana Starobova

**Affiliations:** 1 Medical School, The University of Queensland, Brisbane, QLD, Australia; 2 School of the Environment (Biological Sciences), The University of Queensland, Brisbane, QLD, Australia; 3 Institute for Molecular Bioscience, The University of Queensland, Brisbane, QLD, Australia; 4 Faculty of Medicine and Health, School of Medical Sciences, The University of Sydney, Sydney, NSW, Australia; 5 Mater Centre of Neuroscience, Mater Hospital, Wesley Hospital, The University of Queensland, Brisbane, QLD, Australia; 6 Mater Research Institute-The University of Queensland, South Brisbane, QLD, Australia; 7 The School of Pharmacy and Pharmaceutical Sciences, The University of Queensland, Woolloongabba, QLD, Australia

**Keywords:** biomarkers, chemotherapy-induced peripheral neuropathy (CIPN), genetic polymorphisms, GWAS, neurotoxicity, pharmacogenomics, precision oncology

## Abstract

Chemotherapy-induced peripheral neuropathy (CIPN) is a common, dose-limiting toxicity of cancer treatment, yet reliable biomarkers to predict individual susceptibility remain lacking. Germline genetic variation has been widely investigated as a potential contributor to CIPN risk; however, the consistency and clinical utility of these associations remain unclear. We performed a systematic review of genetic association studies evaluating CIPN across neurotoxic chemotherapies. PubMed, Embase, Web of Science, and the Cochrane Library were searched (April 2025) for human studies assessing germline variants in relation to clinician- or patient-reported CIPN. Eligible studies were identified in four major drug classes: vincristine, taxanes, platinum agents, and bortezomib. Study characteristics, phenotyping approaches, ancestry, and variant-level data were extracted, and study quality was assessed using Q-Genie. Meta-analysis was not performed due to substantial heterogeneity in study design and outcome measures. 72 studies were included, predominantly involving cohorts of European ancestry. The most consistently replicated loci were CEP72 and ETAA1 for vincristine-induced neuropathy; EPHA5, FGD4, and FZD3 for taxane-induced neuropathy; and ABC transporter genes and GSTP1 for platinum-induced neuropathy. For bortezomib, associations involving TRPV1, DNA repair pathways, and inflammatory signalling showed emerging but largely non-replicated evidence. Across studies, marked heterogeneity in CIPN definitions, phenotyping instruments, statistical approaches, and effect allele reporting limited comparability and reproducibility. Collectively, these findings indicate that current pharmacogenomic approaches to CIPN are constrained by poor replication, population bias, and inconsistent methodology, which hinder translation into clinically actionable biomarkers. Future progress will require harmonised phenotyping, multi-ancestry genome-wide studies, and integration of functional validation to enable robust risk stratification and precision oncology approaches to CIPN.

**Systematic Review Registration:**

Identifier PROSPERO [CRD42025635757].

## 1 Introduction

Cancer remains a leading global cause of morbidity and mortality (Wiernik et al., 2024). Despite advances in targeted therapies, traditional chemotherapeutics remain highly effective due to their action on rapidly dividing cells, but it is unclear whether these same mechanisms underlie unintended damage to non-proliferating sensory neurons (Starobova and Vetter, 2017). As a result of recent advances in the early detection of cancer as well as improved survival outcomes (Zajączkowska et al., 2019), the long-term adverse effects of cancer treatment have become increasingly important factors related to an individual’s quality of life (Glare et al., 2014). In some cases, the side effects of cancer treatment can be more disabling than the cancer itself, leading to significant functional impairments that affect both basic and instrumental activities of daily living (Hekmatnia et al., 2022).

Chemotherapy-induced peripheral neuropathy (CIPN) is known as one of the most prevalent and debilitating adverse effects of chemotherapeutic treatments, often manifesting as persistent pain and sensory changes (Brown et al., 2014). Its prevalence varies depending on the agent used, ranging from approximately 19% to over 85% (Fallon, 2013). Specific reported prevalences are particularly high for taxanes (approximately 11%–90%), platinum agents (approximately 19%–90%), vincristine (approximately 30%–90%), and bortezomib (approximately 31%–64%) (Argyriou et al., 2008; Cavaletti and Jakubowiak, 2010; Starobova and Vetter, 2017; Velasco and Bruna, 2015). CIPN presents as a length-dependent sensory neuropathy in a “glove and stocking” pattern, with predominant sensory symptoms causing loss of sensation, gait instability and balance impairment, alongside motor features such as distal weakness (Jones et al., 2015; Park et al., 2017). Analgesic treatment remains largely unsatisfactory and to date, there are no clear well established evidence-based therapies for the prevention and management of CIPN (Sałat, 2020).

It is hypothesized that inter-individual differences in CIPN susceptibility may be genetically determined (Chan et al., 2019). Consistent with this hypothesis, a range of genetic factors, including single-nucleotide polymorphisms (SNPs) involved in drug absorption, distribution, metabolism, and excretion have increasingly been recognized as important contributors to this heterogeneity (Arbitrio et al., 2021). As a result, substantial efforts have focused on identifying genetic variants contributing to CIPN risk beyond the direct toxic effects of chemotherapy itself (Cliff et al., 2017; Seretny et al., 2014).

Genome-wide association studies (GWAS) provide an unbiased framework for uncovering novel loci in contrast to candidate gene studies which typically focus on identifying genetic variants within biologically plausible drug-response pathways (Chua et al., 2009). Despite these genetic findings, replication across studies remains limited, hindered by small sample sizes, differences in genetic ancestry, and inconsistent CIPN grading methods, to name a few, all of which impede the development of robust and reliable genetic biomarkers (Hooshmand et al., 2022).

A well-recognized gap in this literature has been the underreporting of negative or non-replicated genetic associations. Evidence of publication bias has emerged in CIPN research, with studies reporting significant SNP-CIPN associations more likely to be published than those with null or conflicting results (Seretny et al., 2014). This selective reporting of findings may distort the perceived strength and consistency of genetic effects, inflating effect sizes and obscuring the true biological mechanisms underlying CIPN.

This systematic review provides a comprehensive synthesis of genetic associations with CIPN, including both replicated and non-replicated variants. By integrating positive and negative findings, we evaluate the reliability of reported pharmacogenomic signals, identify sources of publication bias, and highlight priority targets for future investigation. Importantly, we move beyond descriptive synthesis to critically examine why these signals have failed to replicate and translate into clinically actionable biomarkers, underscoring the need for validation in larger, genetically diverse cohorts to enable effective personalised CIPN management.

## 2 Methods

This systematic review was conducted and reported in accordance with the Preferred Reporting Items for Systematic Reviews and Meta Analyses guidelines (PRISMA) (Page et al., 2021). This study is a systematic review, and we did not conduct a meta-analysis in view of the broad range of factors included. The protocol was registered prospectively with PROSPERO (registration number CRD42025635757).

### 2.1 Search strategy

**FIGURE 1:**
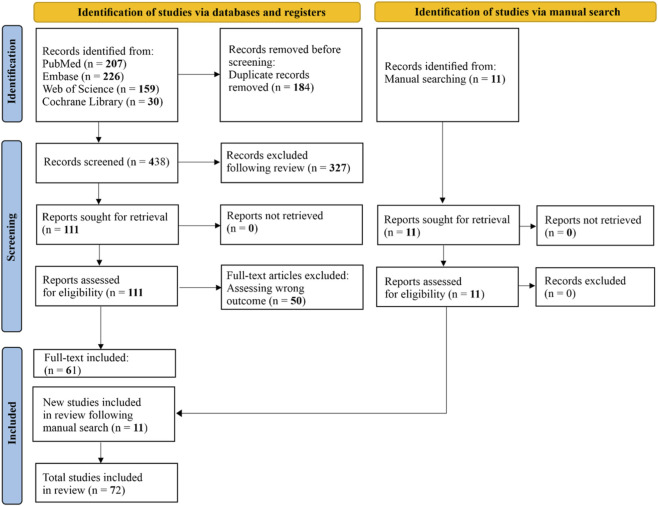
PRISMA flow diagram of the study selection process.

A comprehensive literature search was conducted (database inception in April 2025) by nine independent reviewers across PubMed, Embase, Cochrane Library, and Web of Science, using MeSH (Medical Subject Headings) and Emtree (Excerpta Medica Thesaurus) terms where applicable (Figure 1). The Systematic Review Accelerator’s Polyglot tool was employed to translate PubMed search terms into equivalent terminology for the other databases (Clark et al., 2020). Detailed search strategy and specific terms are provided in Supplementary Material 1. In addition, to ensure the completeness of our search, we cross-checked the peripheral neuropathy-related outcomes in the United Kingdom Biobank GWAS Catalog to identify any potentially missed publications (Sudlow et al., 2015). Each study was reviewed by at least two independent reviewers, the review process was managed using Covidence.

### 2.2 Study selection

Studies were included if published in English, involved patients treated with neurotoxic chemotherapies, and examined genetic associations with neuropathy measured using defined assessment tools. Reviews, meta-analyses, animal studies, conference abstracts and case reports were excluded.

A total of 622 records were identified across four databases, with 184 duplicates removed and 438 screened, leading to 111 full-text articles assessed. 50 studies were excluded for unrelated outcomes, duplicate datasets were cross-checked, and screening was managed using Covidence. Ultimately, 72 studies were included in the final review (Figure 1). Of these, 11 studies (Adjei et al., 2021; Chua et al., 2020; Kanai et al., 2021; Kanai et al., 2016; Klumpers et al., 2022; Komatsu et al., 2015; Mahmoudpour et al., 2018; Martin-Guerrero et al., 2019; Min et al., 2024; Wright et al., 2019; Zgheib et al., 2018) were included by manual search and are catalogued separately in Supplementary Tables. Following screening and eligibility assessment, the included studies were concentrated in four drug classes: vincristine, taxanes, platinum agents, and bortezomib, reflecting the available published evidence.

### 2.3 Quality assessment

The quality of studies identified using database and register search was evaluated using the Quality of Genetic Association Studies (Q-Genie) tool (Sohani et al., 2015; Sohani et al., 2016). Q-Genie was used to assess study quality across key domains including rationale, sample selection, bias, statistics and interpretation, with higher scores indicating better quality. Overall scores ranged from 36 to 67, indicating low to high quality and low to high risk of bias (also see Supplementary Table S1).

### 2.4 Data extraction

The following key information from the identified articles was extracted as part of the review: PubMed identifier, first author, publication year, study characteristics, chemotherapeutic agent, measurement tool for phenotype, phenotype trait reported (such as time to onset of CIPN or severity of CIPN), associated single nucleotide polymorphism (SNP) characteristics (p-value reported, effect size, 95% confidence interval). We extracted the effect allele as well as the effect allele frequency if the study authors clearly mentioned a risk or protective allele in their reporting of a SNP.

To maintain consistency in the reporting in cases where an effect allele was not reported for a SNP, we reported the reference and alternate alleles for the rsID denoting the SNP. Information regarding the reference and alternate alleles was obtained using the SNPNexus tool, using GRCh38 as the reference assembly. We also provided information regarding the reported gene traits to provide additional context (Chelala et al., 2009; Dayem Ullah et al., 2012; Dayem Ullah et al., 2013; Dayem Ullah et al., 2018; Oscanoa et al., 2020). Information regarding gene attribution and reported gene traits were obtained when available from the NIH NCBI database, including the SNP database (dbSNP) based on the closest gene to the relevant SNP.

To minimize the risk of reporting false positive findings in the discovery cohorts of genome-wide association studies, an upper boundary of uncorrected p < 5 × 10^−5^ was set for associated SNPs. In addition, genome-wide significance was defined as the conventional threshold of p < 5 × 10^−8^. For the other study types, a cutoff p < 0.05 was used as the significance threshold.

Formal meta-analysis and statistical tests for publication bias were not undertaken because the included studies lacked sufficient methodological comparability in exposure definitions, phenotyping instruments, and reported effect measures.

## 3 Results

We summarize findings from 72 studies grouped by chemotherapeutic agent, with most conducted in predominantly European populations.

### 3.1 Vincristine

15 studies examined genetic associations with vincristine-induced peripheral neuropathy (VIPN), including 4 GWAS (Broyl et al., 2010; Diouf et al., 2015; Mufti et al., 2024; Yamada et al., 2022) and multiple candidate gene analyses (Aplenc et al., 2003; Egbelakin et al., 2011; Gutierrez-Camino et al., 2018; Sepe et al., 2012; Van De Velde et al., 2022), with additional non-significant discovery entries retained for completeness (Cho et al., 2010; Christofyllakis et al., 2024; Guilhaumou et al., 2011; Kishi et al., 2007; Moore et al., 2011) (for study characteristics see Table 1; for full list of studies see Supplementary Material 2). Most studies assessed VIPN in pediatric cohorts, predominantly those of European origin diagnosed with acute lymphoblastic leukemia. The mean ages of patients included ranged from about 1 year to 66.5 years (Table 1).

**TABLE 1:** Characteristics of vincristine studies included in the review.

Study	Participant age range	Sex distribution	Race/Ethnicity	Cancer type	Drug type	CIPN assessment tool
Mufti et al. (2024)	Pediatric; median age 5.48 years	50.5% males, 49.5% females	Canadian cohort; multi-centre, mixed ancestry	Pediatric cancers	Vincristine	NCI-CTCAE 5.0
Yamada et al. (2022)	66.5 ± 8.0 years	NR	Japanese	Leukemia and malignant lymphoma	Vincristine-containing regimens	Diagnosed by treating physician - no tool specified
Aplenc et al. (2003)	Pediatric; exact range NR	32% males, 68% females	6% African American, 94% other	ALL	Vincristine-containing ALL therapy	CCG Toxicity Criteria (Children’s Cancer Group)
Egbelakin et al. (2011)	Pediatric; ≥1 and ≤18 years	NR	105 Caucasians (6 Hispanic or Latino), 1 African American, and 1 Asian	ALL	Vincristine	NCI-CTCAE 3.0
Sepe et al. (2012)	Pediatric; exact range NR	67.5% males, 32.5% females	White 76.4%, Black 2.5%, Hispanic 15.3%, Asian 2.1%, Other 4.9%	ALL (CCG-1891)	Vincristine-containing ALL therapy	CCG Toxicity Criteria (Children’s Cancer Group)
Kishi et al. (2007)	Pediatric; exact range NR	NR	Race captured by self-report and/or ancestry-informative markers	ALL	ALL therapy including vincristine	NCI-CTCAE 1.0
Diouf et al. (2015)	Pediatric; exact range NR	58.3% males, 41.7% females	Mixed, mainly European	ALL	Vincristine	St. Jude cohort - NCI-CTCAE, COG cohort - Modified Balis scale
van de Velde et al. (2022)	Pediatric, average age 9.17 years	55.56% males, 44.44% females	Mixed, mainly European	Mixed pediatric cancers	Vincristine	NCI-CTCAE and ped-mTNS
Gutierrez-Camino et al. (2018)	Pediatric, average age 5.12 years	58.86% males, 41.14% females	Spanish	B-cell ALL	Vincristine	WHO Peripheral Nervous System toxicity criteria
Christofyllakis et al. (2024)	Adult; 61–80 years	52.9% males, 47.1% females	NR	CD20 positive aggressive B-cell lymphoma	Vincristine-containing lymphoma therapy	NCI-CTCAE 4.3
Yuan et al. (2025)	Pediatric; exact range NR	NR	Chinese	Pediatric ALL	Vincristine	NCI-CTCAE v4.03
Guilhaumou et al. (2011)	Children; 2–16 years	57.7% males, 42.3% females	Caucasian-origin only	Solid tumors	Vincristine	NCI-CTCAE 3.0
Moore et al. (2011)	Children; median age 6.5 years	NR	Australian	Mixed childhood cancers	Vincristine	NCI CTCAE v3.0
Cho et al. (2010)	Adult; 17–80 years	63.8% males, 36.2% females	Korean	Diffuse large B-cell lymphoma	R-CHOP (vincristine-containing)	NCI-CTCAE
Broyl et al. (2010)	Adult; 18–65 years	NR	Mainly European	Newly diagnosed multiple myeloma	Vincristine and bortezomib	NCI-CTCAE 3.0

ALL, Acute Lymphoblastic Leukaemia; NR, not reported.

Firstly, *CEP72* (involved in chromosome segregation) and *ETAA1* (a regulator of replication stress and DNA repair) have been associated with the development of VIPN across GWAS (Diouf et al., 2015) and replicated in an observational pharmacogenetic cohort (Van De Velde et al., 2022) (Table 2). In the discovery GWAS (Diouf et al., 2015), ***CEP72* rs924607** was associated with increased risk of Grade 2–4 VIPN (odds ratio (OR) = 2.43, 95% CI 1.70–3.49; p = 6.33 × 10^−9^), while ***ETAA1* rs17032980** exhibited a similar directional effect (OR = 3.17, 95% CI 1.95–5.17; p = 9.77 × 10^−9^). In a study undertaken by van de Velde et al. (2022), the associations were supported by replication studies in which they reported significant effects on a pediatric-modified Total Neuropathy Score (ped-mTNS) for ***CEP72* rs71585289** (ratio of means = 0.53, 95% CI 0.43–0.66; p < 0.0001) and ***ETAA1* rs35777125** (ratio of means = 0.36, 95% CI 0.19–0.70; p = 0.0007) (Table 2).

**TABLE 2:** Vincristine: SNPs identified in genome-wide association studies (GWAS) and replicated in candidate gene studies (CGS).

rsID	Gene	Reported gene trait	Reference allele	Alternate allele	Effect allele	Effect allele frequency	Sample size	Adjusted p-value	Effect size	Confidence interval	Study name	Study type	CIPN trait studied
rs924607	*CEP72*	Chromosome segregation	C	T	T	36.70%	321	6.33 × 10^−9^	2.43	1.70–3.49	Diouf et al. (2015)	GWAS	Severity (Grade 2–4)
rs71585289	*CEP72*		C	G	NR	NR	90	<0.0001	(Ratio of means) 0.53	(0.43–0.66)	Van De Velde et al. (2022)	Prospective observational pharmacogenetic study within an RCT	
​	*ETAA1*	DNA repair - replication stress protein	A	G	G	19.2%–26.6%	321	9.77 × 10^−9^	3.17	1.95–5.17	Diouf et al. (2015)	GWAS	Severity (Grade 2–4)
rs35777125	*ETAA1*		G	A	NR	NR	90	0.0007	(Ratio of means) 0.36	(0.19–0.70)	Van De Velde et al. (2022)	Prospective observational pharmacogenetic study within an RCT	

Genetic variants associated with vincristine-induced peripheral neuropathy that were identified in GWAS and subsequently replicated in CGS. Columns: rsID and gene as reported in the original publication; reported gene trait extracted from NCBI’s dbSNP annotation; reference and alternate alleles according to SNP Nexus (GRCh38); effect allele and effect allele frequency as reported by the authors; sample size, unadjusted and adjusted P-values, and covariates used for adjustment as stated in the source publication; effect size (odds ratio unless otherwise specified) with 95% confidence interval; study and study type indicating the data source.

In addition to genomic regions being implicated across studies, GWAS have also identified additional candidate variants (Supplementary Table S2). In Diouf et al. (2015), ***MTNR1B*** rs12786200, within a melatonin receptor gene linked to circadian regulation, glucose metabolism, and potentially neuronal stress responses, was associated with reduced VIPN risk (OR = 0.23, 95% CI 0.13–0.40; p = 1.58 × 10^−7^), whereas a cluster of ***TMEM215*** SNPs, including rs4463516, showed elevated risk (OR = 2.89, 95% CI 1.90–4.39; p = 6.83 × 10^−7^). Although the biological function of TMEM215 remains incompletely characterised, transmembrane proteins may influence cellular signalling or membrane homeostasis relevant to neuronal vulnerability.

A more recent GWAS identified multiple loci significantly associated with Grade ≥2 VIPN, including ***MCM3AP*** rs1815857 (OR = 6.25, 95% CI 2.91–12.11; p = 3.13 × 10^−8^), a gene involved in DNA replication and cellular stress responses; ***SPDYA*** rs12474420 (OR = 0.25, 95% CI 0.14–0.43; p = 4.40 × 10^−8^), which regulates cell-cycle progression; ***METTL8*** rs79802223 (OR = 0.17, 95% CI 0.09–0.35; p = 2.46 × 10^−8^), linked to RNA methylation and post-transcriptional regulation; ***PDE4D*** rs12658429 (OR = 0.24, 95% CI 0.14–0.42; p = 4.14 × 10^−8^), which modulates cyclic AMP signalling and inflammatory pathways; ***FBN2*** rs12656510 (OR = 0.36, 95% CI 0.25–0.53; p = 4.06 × 10^−8^), associated with extracellular matrix structure; ***ZFAND3*** rs200858088 (OR = 0.07, 95% CI 0.02–0.21; p = 2.53 × 10^−10^), a zinc-finger protein potentially involved in transcriptional regulation; ***NFIB*** rs10961381 (OR = 0.07, 95% CI 0.02–0.24; p = 2.50 × 10^−8^), a transcription factor implicated in neural development; ***PAPPA*** rs12235805 (OR = 0.20, 95% CI 0.11–0.30; p = 3.04 × 10^−8^), which regulates insulin-like growth factor bioavailability; ***LRRTM3*** rs10997459 (OR = 0.14, 95% CI 0.06–0.32; p = 4.84 × 10^−8^), a neuronal synaptic organiser; and ***NRG3*** rs12253008 (OR = 0.15, 95% CI 0.07–0.33; p = 3.19 × 10^−8^), a member of the neuregulin family involved in neuronal development and Schwann cell signalling. These GWAS-identified loci have not yet been replicated in independent cohorts, underscoring the need for further validation.

Several candidate gene studies have also reported associations between specific variants and VIPN, spanning multiple functional categories (Supplementary Table S3). In a prospective pharmacogenetic study, Van De Velde et al. (2022) identified ***NDRG1*** rs2272653 as protective (OR = 0.49, 95% CI 0.40–0.60; p < 0.0001), consistent with the known role of NDRG1 in myelin maintenance and hereditary neuropathy; ***GARS*** rs1049402 (OR = 0.47, 95% CI 0.34–0.64; p = 0.0013), relevant because mutations in GARS cause axonal Charcot–Marie–Tooth disease; ***FGD4*** rs12823621 (OR = 1.88, 95% CI 1.36–2.59; p < 0.0001), another Charcot–Marie–Tooth gene involved in Schwann cell and cytoskeletal function; and ***SEPTIN9*** rs11650934 (OR = 0.81, 95% CI 0.48–1.38; p < 0.0001), which participates in cytoskeletal organisation and vesicle trafficking (Terrazzino et al., 2015). In a separate study, Gutierrez-Camino et al. (2018) reported that ***miR-3117*** rs12402181 was protective (ratio of means = 0.16, 95% CI 0.05–0.55; adjusted p = 0.0026), *whereas* ***miR-4481*** rs7896283 increased risk (OR = 3.02, 95% CI 1.37–6.69; p = 0.0103) for overall VIPN severity (Gutierrez-Camino et al., 2018). These microRNA loci may influence VIPN susceptibility through post-transcriptional regulation of genes involved in neuronal repair, inflammation, or axonal homeostasis.

Several other discovery variants reported across multiple studies did not reach statistical significance or have not been independently replicated (Broyl et al., 2010; Cho et al., 2010; Christofyllakis et al., 2024; Guilhaumou et al., 2011; Kishi et al., 2007; Moore et al., 2011; Yamada et al., 2022) and are catalogued in Supplementary Table S4.

Taken together, the most consistently replicated signals include ***CEP72*** (chromosome segregation) and ***ETAA1*** (replication stress response) from GWAS, as well as candidate gene associations involving **axonal/neuronal structure** (NDRG1, GARS, FGD4), **miRNA regulation** (miR-3117, miR-4481), and **drug metabolism** (CYP3A4/5).

### 3.2 Taxanes

24 studies investigated the association between genetic variants and taxane-induced peripheral neuropathy (TIPN), including 4 GWAS (Baldwin et al., 2012; Hooshmand et al., 2022; Leandro-García et al., 2013; Sucheston-Campbell et al., 2018) and multiple candidate gene studies across diverse cohorts (see Table 3 for study characteristics, for full list of studies see Supplementary Material 2). Most studies were conducted in adults with breast cancer, although some included older patients (≥65 years) and individuals treated with taxanes for other solid tumors (Table 3). The majority of cohorts were of European ancestry, with few studies conducted in Asian ancestry populations (Nakayama et al., 2024; Tanabe et al., 2020). Reported patient ages ranged from 18 years (De Graan et al., 2013) to 87 years (Leskelä et al., 2011), and sample sizes varied widely, from 25 participants (Sissung et al., 2006) to 1,303 (Abraham et al., 2014).

**TABLE 3:** Characteristics of taxane studies included in the review.

Study	Participant age range	Sex distribution	Race/Ethnicity	Cancer type	Drug type	CIPN assessment tool
Baldwin et al. (2012)	Adult, average age 53.4 years	Females only	Mixed, mainly European ancestry	Primary breast cancer	Paclitaxel	NCI-CTCAE 2.0
Sucheston-Campbell et al. (2018)	Adult, average age 50.1 years	Likely females only	Mixed, European American and African American	Breast cancer	Taxane	CTCAE version 3.0
Hooshmand et al. (2022)	Adult, 27–85 years	4 males, 179 females	NR	Breast 103, ovarian 42, endometrial 25, other 13	Paclitaxel	NCI-CTCAE, TNSc, EORTC QLQ-CIPN20
Vargas-Aliaga et al. (2024)	>18 years	97.7% females	NR	Breast and colorectal cancer	Paclitaxel or oxaliplatin	Presence of chronic CIPN for 6 months (grade ≥1)
Tanabe et al. (2020)	25–81 years	Females only	Japanese	Breast cancer	Paclitaxel/docetaxel	NCI-CTCAE 4.0
Kus et al. (2016)	>18 years	Females only	NR, Turkish	Breast cancer	Paclitaxel or docetaxel	NCI-CTCAE v4.03 + Neuropathic Pain Symptom Inventory questions
Nakayama et al. (2024)	49–80 years	11 males, 14 females	Japanese	Pancreatic cancer	Nab-paclitaxel + gemcitabine	CTCAE 5.0, PRO-CTCAE, FACT/GOG-Ntx
Bergmann et al. (2012)	NR	Females only	Scandinavian caucasian	Ovarian cancer	Paclitaxel and carboplatin	NR
de Jong et al. (2023)	Average age ∼65 years	55.9% males, 44.1% females	Mainly European ancestry	Non-small cell lung cancer	First-line platinum-based therapy and paclitaxel	NCI-CTCAE 4.03
Bosó et al. (2014)	>18 years	Females only	Mixed	Breast cancer	Paclitaxel/docetaxel	NCI-CTCAE 4.0
Hertz et al. (2013)	24–84 years	Females only	European and African American ancestry	Breast cancer	Paclitaxel	NCI-CTCAE 4.0
Sissung et al. (2006)	NR	NR	NR	Advanced solid tumors	Paclitaxel	NCI-CTCAE
de Graan et al. (2013)	18–83 years	52% males, 48% females	Mainly caucasian	Solid tumours	Paclitaxel	NCI-CTCAE 2–4
Sucheston et al. (2011)	23–80 years	Females only	Mainly white	Breast cancer	Paclitaxel	NCI-CTCAE 3.0
Marsh et al. (2007)	>18 years	Females only	Scottish cohort	Ovarian cancer	Carboplatin + taxane	NCI-CTCAE 2.0
Abraham et al. (2014)	>18 years	Females only	European ancestry	Breast cancer	Paclitaxel	NCI-CTCAE 2.0
Leskelä et al. (2011)	Average age 60.7 years	36% males, 64% females	NR, Spanish cohort	Various cancers	Paclitaxel	NCI-CTCAE 2.0
Deng et al. (2023)	38–81 years	Females only	NR, Finnish cohort	Ovarian cancer	Paclitaxel + Carboplatin	NCI-CTCAE 4.0
Abdelfattah et al. (2021)	Average age 49.87 years	Females only	Egyptian	Breast cancer	Paclitaxel	NCI-CTCAE 5.0
Boora et al. (2015)	18–61.6 years	Mainly females	Mainly caucasian	Various cancers	Paclitaxel + Carboplatin	CIPN20
Rizzo et al. (2010)	Average age 57 years	Mainly females	NR	Breast cancer	Taxane (mainly docetaxel)	NCI-CTCAE 3.0
Park et al. (2017)	18–82 years	Females only	Mainly white	Ovarian cancer, ICON7	Paclitaxel	NCI-CTCAE 3.0
Chen et al. (2020)	Average age 52.3 years	Females only	Mainly Caucasian	Breast cancer	Paclitaxel	CIPN-20
Leandro-García et al. (2013)	>18 years	Mixed	White European	Solid tumours	Paclitaxel + carboplatin	NR

NR, not reported; NCI-CTCAE, National Cancer Institute Common Toxicity Criteria for Adverse Events.

A consistent signal of suggestive significance emerged for ***EPHA5* (rs7349683)**, an ephrin receptor gene implicated in axon guidance, which was associated with increased TIPN risk in both GWAS and candidate gene studies (p = 9.6 × 10^−7^; p = 0.03, respectively) (Baldwin et al., 2012; Boora et al., 2015) (Table 4). Baldwin et al. (2012) also identified ***FGD4*** (**rs10771973**; OR = 1.57, p = 2.6 × 10^−6^) alongside ***EPHA5*** (**rs7349683**; OR = 1.63, p = 9.6 × 10^−7^) as suggestively significant loci with known roles in axonal guidance (Supplementary Table S5) (Baldwin et al., 2012). Similarly, Leandro-García et al. (2013) reported ***EPHA6* rs301927** as a risk allele (OR = 2.35, p = 3.44 × 10^−5^), suggesting a potential neuronal targeting mechanism (Supplementary Table S6) (Leandro-García et al., 2013).

**TABLE 4:** Taxane: SNPs identified by GWAS and replicated in candidate gene association studies.

rsID	Gene	Reported gene trait	Reference allele	Alternate allele	Effect allele	Effect allele frequency	Sample size	Unadjusted p-value	Adjusted p-value	Outcomes adjusted for	Effect size	Confidence interval	Study name	Study type	CIPN trait studied
rs7001034	*FZD3*	This gene is a member of the frizzled gene family. The function of this protein is unknown, although it may play a role in Wnt signaling, neurite growth, neural crest development	A	G or T	G/T	0.4	855	3.1 × 10^−9^	​	​	0.57	(0.48–0.69)	Baldwin et al. (2012)	GWAS	Severity
rs7833751	*FZD3*		T	G	G/T	0.4	855	7.5 × 10^−9^	​	​	0.58	(0.49–0.70)	Baldwin et al. (2012)	GWAS	Severity
rs7833751 (a proxy for rs7001034)	*FZD3*		A	G or T	​	(Additive model) 0: 13/58 = 22.4% 1: 20/58 = 34.5% 2: 25/58 = 43.1%	855	​	0.0011	Covariates (cumulative treatment, systemic paclitaxel exposure, and clinical factors)	beta: 0.41 (log-odds scale)	−0.66 −0.17	Baldwin et al. (2012)	GWAS	Severity
rs7349683	*EPHA5*	Belongs to the ephrin receptor subfamily of the protein-tyrosine kinase family. Mediates developmental events, particularly in the nervous system	C	A or T	​	​	855	9.6 × 10^−7^	​	​	1.63	1.34–1.98	Baldwin et al. (2012)	GWAS	Onset
rs7349683			C	A or T	T	0.28	119	0.03	​	​	2.07	1.08–4.10	Boora et al. (2015)	Candidate gene association within a clinical trial	Severity

Genetic variants associated with taxane-induced peripheral neuropathy that were reported in both genome-wide association studies (GWAS) and candidate gene studies (CGS). Columns: rsID and gene as reported in the original publication; reported gene function obtained from NCBI’s dbSNP annotation; reference and alternate alleles based on SNP Nexus (GRCh38); effect allele and effect-allele frequency as reported by the authors; sample size, unadjusted and adjusted P values, and covariates used for adjustment as stated in the source study; effect size (default = odds ratio, unless otherwise specified); 95% confidence interval.

Another significant and replicated finding was **rs7833751** in ***FZD3***, a gene involved in Wnt signaling and neurite development, which was associated with reduced TIPN risk (p = 0.0011 and 7.5 × 10^−9^) (Baldwin et al., 2012; Chen et al., 2020) (Table 2). *SCN9A*, encoding a voltage-gated sodium channel expressed in nociceptive neurons, showed consistent associations across breast cancer cohorts in candidate gene studies (**rs13017637**; OR = 3.46, 95% CI 1.46–8.24, p < 0.005) (Supplementary Table S6) (Tanabe et al., 2020). Another plausible candidate was ***S1PR1*** (**rs74497159)**, which showed suggestively significant association with the risk of developing Grade 2 or higher paclitaxel-induced peripheral neuropathy in a GWAS meta-analysis (p = 3.62 × 10^−7^) (Supplementary Table S11) (Chua et al., 2020).

Across the reviewed studies, a number of key variants in genes related to taxane metabolism and transport were identified (Supplementary Tables S5, S6). For example, variants in ***CYP2C8*** which is a key taxane-metabolizing enzyme, were variably associated with neuropathy risk. ***CYP2C8* rs1058930** increased susceptibility in some studies (hazards ratio (HR) = 1.38, 95% CI 1.03–1.86) (Abraham et al., 2014) but showed inconsistent directionality across other studies (HR range = 0.53–1.46) (Leskelä et al., 2011). Likewise, ***CYP3A5* rs776746** was linked to decreased TIPN risk (HR = 0.51), likely reflecting enhanced taxane clearance (Leskelä et al., 2011). ***CYP1B1* rs1056836**, involved in xenobiotic and estrogen metabolism, conferred modest protection (p = 0.01–0.02; HR = 0.83; OR = 0.81) (Abraham et al., 2014), though replication success varied (Boora et al., 2015; Rizzo et al., 2010).

Transporter genes also featured prominently in the studies that were included in the review (Supplementary Table S6). *ABCB1* variants, including **rs3213619** and **rs1128503**, were associated with altered neuropathy risk, showing consistent or increased effects across independent cohorts (Abraham et al., 2014; Boora et al., 2015; Vargas-Aliaga et al., 2024). ***ABCC2* rs8187710** and ***SLCO1B1* rs3829306** were associated with reduced TIPN risk (p = 0.02; HR = 0.67–0.71), consistent with their roles in systemic drug clearance (Abraham et al., 2014). Conversely, ***TUBB2A* rs9501929** was linked to increased risk (p = 0.005; HR = 1.60), implicating microtubule structural variability in taxane-specific neurotoxicity (Abraham et al., 2014).

Not all the genetic findings were reproducible, and further validation is needed as part of future interdisciplinary clinical-research studies. In particular, the ***ABCB1*** loci **rs2032582** and **rs1128503** showed significant associations in some studies (Abraham et al., 2014; Lambrechts et al., 2015) but failed to replicate or demonstrated variable directionality in others (Leskelä et al., 2011) (Supplementary Tables S5, S6). *ABCB1* encodes P-glycoprotein, a key ATP-dependent drug efflux transporter expressed in the intestine, liver, kidney, blood–nerve barrier, and peripheral neurons, where it may influence systemic taxane exposure, tissue drug accumulation, and neuronal susceptibility to toxicity. The inconsistent findings therefore likely reflect heterogeneity across study populations, treatment regimens, ancestry, and phenotyping methodologies.

Several other discovery variants reported across multiple research studies did not reach statistical significance or have not been independently replicated and are catalogued in Supplementary Table S7.

### 3.3 Platinum-based agents

19 studies investigated the genetic associations with platinum-induced peripheral neuropathy (PIPN) including 1 GWAS (Dolan et al., 2017) as well as multiple candidate gene studies (See Table 5 for study characteristics, for full list of studies see Supplementary Material 2). The GWAS undertaken by Dolan et al. (2017) identified **rs7349683** within ***EPHA5***, a gene implicated in normal neuronal development as well as in the repair mechanisms activated after nerve injury (Dolan et al., 2017). However, none of the SNPs reported in this GWAS were subsequently replicated in candidate gene analyses. This represents an important limitation of previous research, given the scarcity of GWAS specifically investigating PIPN. Most platinum-related studies have focused on other chemotherapy-related toxicities (e.g., neutropenia), which did not meet the exclusion criteria for this review.

**TABLE 5:** Characteristics of platinum derivates studies included in the review.

Study	Participant age range	Sex distribution	Race/Ethnicity	Cancer type	Drug type	CIPN assessment tool
Katayanagi et al. (2019)	35–81 years	Mostly males	Japanese	Colorectal, pancreatic and gastric cancers	Platinum-based antitumour therapy	NCI-CTCAE v4.0
De Jong et al. (2023)	Average age ∼65 years	55.9% males, 44.1% females	Mainly European ancestry	Non-small cell lung cancer	First-line platinum-based therapy and paclitaxel	NCI-CTCAE 4.03
Johnson et al. (2015)	NR	NR	NR	Primary lung cancer	Cisplatin, Carboplatin	NCI-CTCAE
Hong et al. (2011)	37–74 years	71.2% males, 28.8% females	NR	Metastatic colorectal cancer	S-1 + oxaliplatin	NCI-CTCAE 3.0
Lecomte et al. (2006)	24–84 years	52 males, 38 females	Mainly caucasian	Gastrointestinal solid tumours	Oxaliplatin-based chemotherapy	Oxaliplatin-specific scale reported by Caussanel et al.
Custodio et al. (2014)	23–85 years	51.4% males, 48.6% females	NR	Stage II–III colon cancer	Oxaliplatin + fluoropyrimidine adjuvant chemotherapy	NCI-CTCAE 2.0
Terrazzino et al. (2015)	>18 years	Mixed, NR	Caucasian	Colorectal cancer	Oxaliplatin-based chemotherapy	TNSc, NCI-CTCAE 3.0
Chen et al. (2010)	>18 years	61.6% males, 38.4% females	Chinese	Metastatic colorectal carcinoma	FOLFOX-4	NCI-CTCAE
Kumamoto et al. (2013)	32–84 years	41 males, 22 females	NR	Metastatic colorectal cancer	First-line mFOLFOX6	NCI-CTCAE 3.0
Cecchin et al. (2013)	25–82 years	57% males, 43% females	NR	Adjuvant colorectal cancer	FOLFOX4	NCI-CTCAE 2.0, Oxaliplatin-specific scale reported by Caussanel et al.
Won et al. (2012)	24–75 years	182 males, 161 females	NR	Colon cancer	Oxaliplatin-based chemotherapy	NCI-CTCAE 3.0
Vargas-Aliaga et al. (2024)	>18 years	47 male/105 female	NR	Breast and colorectal cancer	Paclitaxel or oxaliplatin	Presence of chronic CIPN for 6 months (grade ≥1)
Ruzzo et al. (2014)	>18 years	57.6% males, 42.4% females	NR	High-risk colon cancer, TOSCA trial	Adjuvant oxaliplatin + fluoropyrimidines	NCI-CTCAE 2.0
Lai et al. (2009)	>18 years	55.1% males, 44.9% females	Asian patients	Colorectal carcinoma	FOLFOX-4	NCI-CTCAE
Chua et al. (2009)	31–75 years	68% males, 32% females	Mainly caucasian	Colorectal cancer	FOLFOX	NCI-CTCAE
Antonacopoulou et al. (2010)	>18 years	Mixed, NR	NR	Colorectal cancer	Oxaliplatin	TNSc
Kanai et al. (2010)	41–80 years	62% males, 38% females	Japanese	Colorectal cancer	FOLFOX6	Oxaliplatin-specific scale reported by Caussanel et al.
McLeod et al. (2010)	19–88 years	62% males, 38% females	Mostly white	Metastatic colorectal cancer, N9741	Oxaliplatin-based regimens	NCI-CTCAE 2.0
Kweekel et al. (2009)	37–81 years	∼50%/50% depending on variant	Mainly caucasian	Advanced colorectal cancer	Oxaliplatin	NCI-CTCAE 2.0

NR, not reported; NCI-CTCAE, National Cancer Institute Common Toxicity Criteria for Adverse Events; TNS, Total Neuropathy Score.

Most of the studies that met the inclusion criteria enrolled individuals of European ancestry who were primarily diagnosed with colorectal cancer (Table 5). There were some studies which included participants of East Asian ancestry although some of the studies did not specifically report any ancestry-based clinical information. The patient ages ranged from 18 years (Dolan et al., 2017) to 88 years (Cecchin et al., 2013), with a mean age of approximately 65 years across studies (Table 5).

Supplementary File 1 summarizes SNPs identified in candidate gene association studies. Although candidate gene approaches are inherently more prone to bias than hypothesis-free GWAS, the variants that were identified through this method have been implicated across a number of important pathophysiological processes that are involved in the development of CIPN. However, further functional validation is needed as part of future translational studies.

The most extensively studied variant in platinum-induced peripheral neuropathy was ***GSTP1* rs1695**, which showed marked inconsistency across studies (Supplementary File 1). *GSTP1* encodes glutathione S-transferase Pi 1, a key phase II detoxification enzyme involved in conjugation of reactive electrophilic compounds with glutathione relevant to platinum derivates metabolism. Reported effect estimates varied substantially, ranging from protective (HR = 0.47, 95% CI 0.21–1.04, p = 0.03 (Hong et al., 2011)) to increased risk (OR = 6.08, 95% CI 1.15–32.18, p = 0.05 (Kumamoto et al., 2013)), with some studies reporting no significant association (Chen et al., 2020; Hong et al., 2011; Katayanagi et al., 2019; Kumamoto et al., 2013; Lecomte et al., 2006). These discrepancies were observed across cohorts differing in cancer type, treatment regimen, and phenotype definition. Specifically, studies varied in the use of oxaliplatin-based combinations (e.g., FOLFOX), outcome measures (cumulative, severe, or chronic CIPN), grading instruments (different CTCAE versions, TNSc, or study-specific scales), and timing of assessment (Chen et al., 2020; Hong et al., 2011; Katayanagi et al., 2019; Kumamoto et al., 2013; Lecomte et al., 2006). Together, these findings indicate that the direction and magnitude of the ***GSTP1*** **rs1695** association are not consistent across studies.

In a prospective candidate gene association undertaken by De Jong et al. (2023), the authors found a significant association between ***TRPV1* rs879207** and the severity of PIPN (De Jong et al., 2023). *TRPV1* encodes a nociceptive ion channel involved in pain signalling, thermal sensitivity, and calcium-dependent neuronal excitability, making it a biologically plausible mediator of chemotherapy-induced neuropathic pain and sensory dysfunction. The G allele was linked to increased neuropathy severity (OR 5.2, 95% CI 2.1–12.8; p = 0.012) in a cohort of 320 patients. The TRPV1 protein encodes a non-selective cation channel that acts as the receptor for capsaicin and plays a key role in nociception and pain signaling (Rosenbaum and Simon, 2007).

Multiple studies have implicated ATP-binding cassette (ABC) transporter family genes as contributing to the susceptibility of oxaliplatin-induced peripheral neuropathy (Yang et al., 2021). Variants across ***ABCC1*** *,* ***ABCC2***, and ***ABCC4*** have been associated with differences in neuropathy severity, supporting a role for altered drug efflux and detoxification mechanisms in mediating neurotoxicity (Supplementary File 1). Two polymorphisms in ***ABCC4*, rs1729786** and **rs6492763**, were associated with opposing effects on neuropathy severity, with **rs1729786** conferring a protective effect (OR 0.63, 95% CI 0.45–0.87; adjusted p = 0.006) and **rs6492763** associated with increased risk (OR 1.42, 95% CI 1.02–1.98; adjusted p = 0.0406) (Johnson et al., 2015). In ***ABCC1*, rs2074087** and **rs35587** were both linked to reduced neuropathy severity, with effect estimates ranging from OR 0.43 (95% CI 0.22–0.86; p = 0.017) to OR 0.47 (95% CI 0.23–0.96; p = 0.0375) (Cecchin et al., 2013).

Several ***ABCC2*** polymorphisms were significantly associated with increased levels of neuropathy severity, including **rs1885301** (OR 3.06, 95% CI 1.35–6.92; p = 0.0072), **rs717620** (OR 14.39, 95% CI 1.63–127.02; p = 0.0164), **rs4148396** (OR 4.69, 95% CI 1.60–13.74; p = 0.0048), and **rs3740066** (OR 2.99, 95% CI 1.16–7.70; p = 0.0231). In contrast, **rs2273697** was found to have a protective association (OR 0.44, 95% CI 0.20–0.98; p = 0.0043) (Cecchin et al., 2013). Collectively, these findings highlight the importance of ABC transporter polymorphisms, particularly within ***ABCC2***, as potential modulators of platinum drug clearance as well as neuronal toxicity. The direction of effects across variants and the lack of replication across independent cohorts warrant further validation across larger, ethnically diverse populations.

Several single-study associations implicated genes involved in DNA repair pathways. The alkyl-adduct reversal enzyme ***MGMT*** exhibited a protective effect at **rs11016884** (OR = 0.64, 95% CI 0.46–0.90, p = 0.0104) (Johnson et al., 2015). Within the nucleotide excision repair pathway, ***ERCC1* rs11615** conferred increased neuropathy risk (OR = 2.4, 95% CI 1.1–5.4, p = 0.047) (Chen et al., 2010). The homologous recombination mediator ***RAD51*** carried a protective allele at **rs3092981** (OR = 0.59, 95% CI 0.35–0.99, p = 0.0445) (Johnson et al., 2015), whereas the mismatch repair component ***MSH3* rs26279** was associated with greater neuropathy risk (OR = 1.39, 95% CI 1.01–1.93, p = 0.0467) (Johnson et al., 2015). These findings highlight the involvement of multiple DNA repair mechanisms, including nucleotide excision repair, homologous recombination, and mismatch repair, as biologically plausible pathways contributing to platinum-induced neurotoxicity (Supplementary File 1).

Several other discovery variants reported across multiple studies did not reach statistical significance or have not been independently replicated and are catalogued in Supplementary Table S8.

### 3.4 Bortezomib

Six studies were identified which specifically investigated the association between genetic polymorphisms and the risk of developing bortezomib-induced peripheral neuropathy (BIPN). The identified studies included 3 GWAS (Campo et al., 2018; García-Sanz et al., 2017; Magrangeas et al., 2016), 1 GWAS replication cohort (Campo et al., 2017), and two candidate gene studies (Corthals et al., 2011; Zhang et al., 2024) (see Table 6 for study characteristics, for full list of studies see Supplementary Material 2). The relatively small number of bortezomib studies reflects a limited pharmacogenomic evidence base for this drug class rather than a search strategy constraint, as no restrictions on chemotherapeutic agents were applied (Supplementary Material). All studies were conducted in adult populations with multiple myeloma. Most of the cohorts included individuals predominantly of European ancestry although there was one study which only included individuals of Chinese ancestry (Zhang et al., 2024). Two studies (García-Sanz et al., 2017; Magrangeas et al., 2016) did not specify the ancestry of the participants but recruited individuals from the GEM05MAS65, HOVON-65, and IFM trials, which have been reported elsewhere and primarily include patients of European descent.

**TABLE 6:** Characteristics of bortezomib studies included in the review.

Study	Participant age range	Sex distribution	Race/Ethnicity	Cancer type	Drug type	CIPN assessment tool
Magrangeas et al. (2016)	NR	NR	European ancestry	Multiple myeloma	Bortezomib	NCI-CTCAE 3.0
García-Sanz et al. (2017)	>18 years	Mixed, NR	European ancestry	Multiple myeloma	Bortezomib and/or thalidomide	NCI-CTCAE 3.0
Campo et al. (2018)	>18 years	Mixed, NR	German cohort; exact race/ethnicity NR	Multiple myeloma	Bortezomib	NR (Peripheral Neuropathy Grades 2–5)
Broyl et al. (2010)	18–65 years	PAD arm: 247 males, 164 females; VAD arm: 253 males, 163 females	European ancestry	Newly diagnosed multiple myeloma	Bortezomib-containing PAD vs. vincristine-containing VAD	NCI-CTCAE 3.0
Zhang et al. (2024)	Average > 60 years	114 males, 90 females	Chinese	Multiple myeloma	Bortezomib-based therapy	NCI-CTCAE 5.0
Corthals et al. (2011)	31–65 years	∼60% males, 40% females depending on cohort	Mainly European ancestry	Multiple myeloma	Bortezomib	NCI-CTCAE 3.0

NR, not reported; NCI-CTCAE, National Cancer Institute Common Toxicity Criteria for Adverse Events.

None of the SNPs identified in the GWAS setting achieved statistical significance upon replication in candidate gene studies (Table 7). Nonetheless, several variants have been highlighted in candidate gene analyses, including ***MTHFR*** (rs1801131), which regulates folate metabolism and methylation pathways important for neuronal repair and oxidative stress responses; ***EDN1*** (rs5370), encoding endothelin-1, a potent vasoactive peptide implicated in microvascular dysfunction and neuroinflammation; ***IL17RD*** (rs1545981), a modulator of interleukin-17 and inflammatory signalling pathways; and the ABC transporter gene ***ABCC6*** (rs8058696), which may influence xenobiotic transport and tissue susceptibility to toxic metabolites. These loci demonstrated significant associations with BIPN across several independent studies. The odds ratios ranged from 1.58 (***EDN1***) to 2.63 (***MTHFR***), with rs1801131 achieving p < 0.001 in a Chinese Han cohort (Zhang et al., 2024), which was further supported by findings from a Dutch clinical cohort study (Broyl et al., 2010). However, replication findings for ***EDN1*** and ***IL17RD*** differed in effect direction, further highlighting the potential impact of population or phenotype heterogeneity (Supplementary Table S9) (Campo et al., 2017).

**TABLE 7:** Bortezomib: SNPs reported by multiple GWAS, pending replication in candidate gene association studies.

rsID	Gene	Reported gene trait	Reference allele	Alternate allele	Effect allele	Sample size	Unadjusted p-value	Adjusted p-value	Outcomes adjusted for	Effect size	Confidence interval	Study name	Study type	CIPN trait studied
rs2839629	*PKNOX1*	Modulates transcriptional activity of chemokine MCP-1 gene	G	A	A	429	​	0.036	Family-wise error rate correction	2.04	(1.11–3.33)	Magrangeas et al. (2016)	GWAS	Severity, grade 2 or greater
rs80146384	*PRDM6*	Protein encoded by this gene is a transcriptional repressor and a member of the PRDM family. The encoded protein is involved in regulation of vascular smooth muscle cells (VSMC) contractile proteins	A	G	​	172	7.21E−06	0.1305	False Discovery rate by the Benjamini–Hochberg method	​	​	García-Sanz et al. (2017)	GWAS	Severity
rs6552496	*TENM3-AS1, LINC02500*	Binding site for transcriptional repressors Foxo, Gfi, and Gfi1b	C	A	C	646	7.82 × 10^−6^	​	​	2	1.46–2.76	Campo et al. (2018)	GWAS	Severity
rs17748074	*DCC*	Encodes a netrin 1 receptor. Mediates axon guidance of neuronal growth cones towards sources of netrin 1 ligand. The protein functions as a tumor suppressor, and is frequently mutated or downregulated in colorectal cancer and esophageal carcinoma	A	G or T	A	646	8.60 × 10^−6^	​	​	1.96	1.45–2.65			

Single-nucleotide polymorphisms (SNPs) associated with bortezomib-induced peripheral neuropathy which were identified through genome-wide association studies (GWAS) but not yet replicated in candidate gene analyses. Columns: rsID and Gene as reported in the original study; Reported gene function extracted from NCBI’s dbSNP gene annotation; Reference allele/Alternate allele taken from SNP Nexus (GRCh38); Effect allele and Effect-allele frequency as stated by the authors, Sample size, Unadjusted p-value, Adjusted p-value, and Outcomes adjusted for, taken directly from the publication; Effect size defaults to odds ratio unless otherwise indicated; Confidence interval, Study, and Study type indicate the source.

In terms of the GWAS findings, ***PKNOX1*** rs2839629 was associated with an increased risk of grade ≥2 neuropathy (OR = 2.04, 95% CI 1.11–3.33; p = 0.036) (Magrangeas et al., 2016). ***PKNOX1*** encodes a transcription factor involved in gene regulation during neuronal development and inflammatory responses, supporting a plausible role in BIPN susceptibility. Additional loci included ***PRDM6*** rs80146384, a regulator of vascular smooth muscle differentiation and vascular homeostasis (García-Sanz et al., 2017), as well as ***TENM3-AS1/LINC02500*** rs6552496 and ***DCC*** rs17748074, both linked to neuronal development and axon guidance pathways (Campo et al., 2018). The latter GWAS also identified ***CDH13*** rs8060632, encoding a cadherin involved in cell adhesion and neuronal connectivity, and ***CMYA5*** rs12521798, a cytoskeletal-associated gene potentially relevant to structural resilience, surpassing suggestive thresholds, though none reached genome-wide significance or were validated in independent cohorts (Table 7).

Several additional variants remain single-study findings, including ***MTHFR*** rs1801133 and rs17421511 (Zhang et al., 2024), and ***ABCC1*** rs2014800 (Campo et al., 2018), where ***ABCC1*** encodes a multidrug resistance transporter involved in cellular efflux of xenobiotics and oxidative metabolites. Previously proposed associations, such as intronic variants in ***RHOBTB2***, a regulator of cytoskeletal dynamics and vesicle trafficking, and ***PLCG2*** rs45443101, involved in intracellular calcium and immune signalling, were not replicable upon reanalysis (Broyl et al., 2010; García-Sanz et al., 2017).

Collectively, these results indicate biologically plausible roles for genes involved in axon guidance, vascular signalling, detoxification transport, folate metabolism, and inflammatory regulation, but underscore the lack of consistent replication across studies and the need for larger, multi-ancestry GWAS and functional validation efforts (Tables 4; Supplementary Tables S9, S10).

Several recently discovered variants that were reported across multiple studies did not reach statistical significance and were not independently replicated and are catalogued in Supplementary Table S10.

### 3.5 Additional studies included following manual search

Across taxanes, vinca alkaloids, and platinum-based therapies, 11 manually curated studies were also included in this review (Adjei et al., 2021; Chua et al., 2020; Kanai et al., 2021; Kanai et al., 2016; Klumpers et al., 2022; Komatsu et al., 2015; Mahmoudpour et al., 2018; Martin-Guerrero et al., 2019; Min et al., 2024; Wright et al., 2019; Zgheib et al., 2018). For a comprehensive summary of significant and non-significant findings from these studies see Table 8 for study characteristics and Supplementary Tables S11, S12.

**TABLE 8:** Characteristics of studies included in the manually curated dataset.

Study	Participant age range	Sex distribution	Race/Ethnicity	Cancer type	Drug type	CIPN assessment tool
Chua et al. (2020)	>18 years	NR	Meta-analysis included European and Asian ancestry cohorts	Mixed solid tumours	Microtubule-targeting agents (paclitaxel, docetaxel, ixabepilone, eribulin, vincristine)	NCI-CTCAE
Wright et al. (2019)	2–18 years	53 males, 42 females	Mixed	Pediatric acute lymphoblastic leukemia	Vincristine	Total Neuropathy Score-Pediatric Vincristine (TNS-PV)
Adjei et al. (2021)	Median 56 years	Mainly females	Mainly white	Mixed cancers (Alliance/NCCTG prevention cohorts)	Paclitaxel, carboplatin, oxaliplatin	QLQ-CIPN20
Klumpers et al. (2022)	0–24 years	74 males, 65 females	Dutch cohort	Brain tumours	Vincristine	NCI-CTCAE 4.03
Zgheib et al. (2018)	1–18 years	117 males, 103 females	Arab children (Lebanese cohort)	Acute lymphoblastic leukemia	Vincristine	NCI-CTCAE
Kanai et al. (2021)	20–80 years	546 males, 478 females	Japanese	Stage II/III colon cancer	Oxaliplatin (adjuvant)	DEB-NTC NCI-CTCAE
Kanai et al. (2016)	20–80 years	517 males, 507 females	Japanese	Colon cancer	Oxaliplatin (JOIN Trial)	DEB-NTC + NCI-CTCAE 4.0
Min et al. (2024)	40–79 years	172 males, 138 females	South Korean, East Asian	Multiple myeloma	Bortezomib	DN4 questionnaire
Mahmoudpour et al. (2018)	Average age ∼57 years	58% males, 42% females	European ancestry	Multiple myeloma	Bortezomib, vincristine	NCI-CTCAE 2.0
Martin-Guerrero et al. (2019)	1–18 years	64 males, 56 females	Spanish, European ancestry	Pediatric acute lymphoblastic leukemia	Vincristine	NCI-CTCAE
Komatsu et al. (2015)	US cohort: average 54.2 years Japanese cohort: average 57.8 years	Mainly females depending on cohort	European American vs. Japanese comparative cohorts	Breast cancer	Paclitaxel	NCI-CTCAE

NR, not reported; NCI-CTCAE, National Cancer Institute Common Toxicity Criteria for Adverse Events; DEB-NTC, DEB Neurotoxicity Criteria (patient reported); DN4, Douleur Neuropathique en 4 Questions.

Paclitaxel-associated CIPN showed the strongest and most numerous associations within this data additional set, including genome-wide significant loci and variants in genes involved in neuronal structure, cytoskeletal regulation, transcriptional control, and immune signaling (Adjei et al., 2021; Chua et al., 2020; Mahmoudpour et al., 2018). Notably, rs74497159 in ***S1PR1*** emerged as a robust paclitaxel-specific association, implicating sphingosine-1-phosphate signaling, an established regulator of endothelial function, neuroinflammation, and barrier integrity, as a mechanistically plausible and potentially druggable pathway in CIPN (Chua et al., 2020).

For vincristine, fewer loci were reported, however similar to studies above, the most consistent evidence was observed for variants in ***CEP72***, a gene involved in chromosome segregation, across multiple cohorts and genetic models. Additional associations implicated genes related to purine metabolism, and inflammatory signaling, although findings were generally derived from smaller candidate gene studies (Klumpers et al., 2022; Mahmoudpour et al., 2018; Wright et al., 2019).

In oxaliplatin-treated cohorts, GWAS identified multiple loci associated with neuropathy severity and recovery, mapping to genes involved in synaptic transmission, calcium signaling, metal transport, extracellular matrix integrity, and neuronal development (Kanai et al., 2021). Several associations localized to non-coding regions, suggesting regulatory mechanisms contributing to inter-individual variability in both CIPN onset and resolution.

However, comparison of Supplementary Tables S11, S12 revealed additional substantial heterogeneity and inconsistency in these reported genetic associations with CIPN across studies and treatment regimens. Variants reported as significant in some cohorts frequently showed null associations, opposite directions of effect, or lack of independent replication in others.

For vincristine, ***CEP72*** exemplified this discordance: while certain studies reported significant associations with neuropathy severity (Supplementary Table S11), independent analyses of **rs868649** and **rs924607** showed no association (Supplementary Table S12) (Klumpers et al., 2022; Zgheib et al., 2018).

For paclitaxel, several loci highlighted in Supplementary Table S11, including genes involved in cytoskeletal and neuronal processes (e.g., ***FGD4***), demonstrated weaker or non-significant effects in curated null studies, and multiple previously proposed candidates in drug metabolism and transport pathways (***CYP2C8*, *ABCC2*, *ABCB1***) failed to reach corrected significance thresholds (Supplementary Table S12) (Adjei et al., 2021; Komatsu et al., 2015).

In oxaliplatin-treated cohorts, variants in detoxification, DNA repair, and ion channel pathways (e.g., ***GSTP1*, *ERCC1*, *SCN10A***) were predominantly non-significant across multiple studies (Supplementary Table S12) (Adjei et al., 2021; Kanai et al., 2016). Similarly, for bortezomib, the majority of candidate variants spanning immune signaling, proteasome components, and transporter genes showed null associations (Supplementary Table S12) (Min et al., 2024).

## 4 Discussion

Chemotherapy-induced peripheral neuropathy (CIPN) remains a major dose-limiting toxicity of cancer treatment, with prevalence ranging from approximately 19% to over 85% depending on the chemotherapeutic agent (Fallon, 2013; Starobova and Vetter, 2017). Despite its clinical significance and long-term impact on quality of life, there are currently no effective disease-modifying therapies (Sałat, 2020). This systematic review synthesised evidence from 72 studies investigating germline genetic contributions to CIPN across vincristine, taxanes, platinum agents, and bortezomib. While numerous genetic associations have been reported, including replicated loci such as *CEP72* and *ETAA1* in vincristine-induced neuropathy (Diouf et al., 2015; Van De Velde et al., 2022) and *EPHA5*, *FGD4*, and *FZD3* in taxane-induced neuropathy (Baldwin et al., 2012; Boora et al., 2015; Chen et al., 2020; Leandro-García et al., 2013; Tanabe et al., 2020) the overall evidence is characterised by limited reproducibility, inconsistent directionality of effects, and lack of clinical translation. Importantly, by integrating both positive and negative findings, this review moves beyond descriptive synthesis to critically evaluate the reliability of pharmacogenomic signals in CIPN and identifies the key barriers preventing translation into clinically actionable biomarkers.

### 4.1 Why pharmacogenomic findings in CIPN fail to replicate

Although numerous variants have been proposed, only a small subset has demonstrated consistent replication across independent cohorts, and none has yet achieved sufficient clinical validity for predictive implementation. Collectively, the available evidence supports the biological plausibility of several pathophysiological pathways involved in CIPN but also highlights substantial methodological limitations that hinder reproducibility and translation into clinical practice. Additionally, it is important to note that different chemotherapeutic agents act through distinct mechanisms, making genetic heterogeneity in CIPN risk factors likely.

The variability observed for ***GSTP1 rs1695*** highlights a key limitation in CIPN pharmacogenomics. Despite being the most frequently studied variant in platinum-induced neuropathy, its effect direction is not stable across studies (Chen et al., 2020; Hong et al., 2011; Katayanagi et al., 2019; Kumamoto et al., 2013; Lecomte et al., 2006). This inconsistency appears to be driven by substantial heterogeneity in study design (Supplementary Table S13), including differences in cancer type, platinum regimen and cumulative exposure, phenotype definition (e.g., cumulative *versus* chronic neuropathy), assessment tools, and timing of evaluation (Chen et al., 2020; Hong et al., 2011; Katayanagi et al., 2019; Kumamoto et al., 2013; Lecomte et al., 2006). Population differences may further contribute, given variability in allele frequencies and genetic background across cohorts. As a result, ***GSTP1 rs1695*** is best interpreted as a context-dependent association, rather than a robust or generalisable biomarker. This finding underscores a broader issue in the field: biologically plausible pathways, such as detoxification and oxidative stress, are repeatedly implicated, but variant-level effects remain inconsistent due to unresolved heterogeneity in phenotyping and clinical context.

A major limitation to replication was substantial heterogeneity in study design, with CIPN assessed using diverse clinical scales and patient-reported measures, limiting comparability and increasing measurement error. Statistical methods varied widely, including different multiple-testing corrections or nominal p-values, and many studies inadequately adjusted for confounders, further reducing interpretability. Inconsistent allele annotation frequently complicated interpretation. In many reports, the effect allele was not explicitly specified, preventing harmonization and sometimes reversing inferred effect directions. As highlighted by Wootton and Sallis, (2020), inconsistent terminology, such as “reference”, “alternate”, “minor”, or “A1/A2”, without clarification, undermines data integrity and limits reproducibility. Entries lacking clear annotation were coded as “Not reported” in this review, reflecting broader issues in pharmacogenomic reporting practices.

Another major limitation of the current evidence base is the strong overrepresentation of European ancestry populations, particularly in taxane studies where 19 of 25 cohorts were predominantly European (Table 3). This is not a minor limitation but a critical barrier to generalisability. Allele frequencies for several commonly studied variants differ substantially across populations, and effect sizes estimated in European cohorts may not translate to non-European groups. For example, variants such as *CYP3A5*, which is rare in European populations but common in African ancestry groups, and loci including *EPHA5, FGD4*, and *S1PR1*, identified primarily in European cohorts, may have different effect sizes or linkage structures in other populations. Similarly, *CEP72* (vincristine) and *GSTP1 rs1695* (platinum agents) show population-dependent allele frequencies that may influence both statistical power and observed direction of effect.

The limited representation of Asian, African, and other ancestries restricts the ability to determine whether observed associations are broadly applicable or population-specific. As a result, current pharmacogenomic findings cannot be assumed to be generalisable across global patient populations. This has direct implications for precision oncology, where applying European-derived genetic risk markers to diverse populations may lead to inaccurate risk prediction. Addressing this limitation will require multi-ancestry cohorts, replication in underrepresented populations, and explicit reporting of ancestry-stratified effects.

Cumulative chemotherapy exposure represents another source of heterogeneity across CIPN pharmacogenomic studies. As summarised in Supplementary Table S14, adjustment for cumulative dose was inconsistently performed or reported across the included studies. This limitation is particularly relevant for taxane- and oxaliplatin-treated cohorts, where neuropathy severity and persistence are closely related to cumulative exposure. In unadjusted analyses, a variant that appears protective may instead identify patients who tolerated higher cumulative doses before developing neuropathy, whereas a risk variant may be confounded by treatment modification, early discontinuation, or differential exposure. Therefore, inconsistent dose adjustment likely contributes to non-replication and unstable effect directions across studies. Future pharmacogenomic studies should report planned and delivered cumulative dose, dose reductions, treatment delays, and discontinuation, and should include these exposure variables in sensitivity analyses where possible.

A further important and underappreciated source of heterogeneity across studies is the wide age range of included patient populations (Tables 1, 3, 5, 7). The studies reviewed span pediatric cohorts, particularly in vincristine-treated acute lymphoblastic leukemia, to older adult populations receiving taxanes or platinum-based therapies, with reported ages ranging from early childhood to over 85 years. These populations differ fundamentally in pharmacokinetics, drug metabolism, neuronal resilience, and comorbidity burden, all of which are known to influence CIPN risk and severity (Starobova and Vetter, 2017; Tay et al., 2022). As a result, genetic associations identified in pediatric settings may not be directly comparable to those observed in adult or elderly populations. For example, variants influencing drug metabolism or neurodevelopmental pathways in children may have different functional consequences in the context of age-related neuronal vulnerability or cumulative physiological stress in older adults. Despite this, relatively few studies explicitly account for age as a modifier or perform age-stratified analyses. The absence of systematic consideration of age effects likely contributes to inconsistencies in the direction and magnitude of reported associations across studies. This highlights the need for future pharmacogenomic investigations to incorporate age as a key biological variable, including through stratified analyses and multi-cohort validation across distinct age groups.

Interpretation and reproducibility is also complicated by numerous variants of uncertain significance (VUS) requiring combined clinical, computational and functional evidence, with unclear contributions to CIPN risk. Integrative functional and population analyses are needed to establish pathogenicity, although many VUS will remain unresolved.

Additionally, small discovery cohorts with inflated effect estimates that fail replication point to a “winner’s curse,” and many reported variants lie in non-coding regions with unknown functional consequences, underscoring the need for integrative functional genomics.

Although publication bias is a recognized concern in CIPN pharmacogenomics, formal quantitative assessment (e.g., funnel plots or Egger’s regression) was not feasible because studies differed substantially in chemotherapy exposures, phenotype definitions, genetic models, and reporting metrics. Nevertheless, the literature demonstrates patterns consistent with selective reporting and winner’s curse, whereby numerous loci were significant only in discovery cohorts and showed attenuated, null, or inconsistent effects in later studies.

To address whether replication failure in CIPN pharmacogenomics is primarily methodological or biological, we incorporated Q-Genie scores into interpretation and stratified findings by study quality (Table 9). Overall, 52.4% of studies were high quality and 37.7% moderate quality. Higher-quality studies were typically GWAS, prospective cohorts, or multicentre analyses, and identified the most credible replicated loci, including ***CEP72*** and ***ETAA1*** (vincristine), ***EPHA5*** *,* ***FGD4*** *,* ***FZD3*** *,* ***SCN9A*** *,* and ***S1PR1*** (taxanes), and recurrent ***GSTP1*** and ***ABCC2*** associations in platinum-treated cohorts. Importantly, Table 9 shows that non-replication was not limited to lower-quality studies. Several high-quality studies also reported null validation attempts, reduced effect sizes, or inconsistent directions of effect, particularly for ***ABCB1*** *,* ***CYP2C8*** *,* ***CYP3A5*** *,* ***GSTP1***, and multiple GWAS loci. Thus, improved methodology alone does not fully explain reproducibility failures. Moderate-quality studies showed mixed positive and null findings, while lower-quality studies more often reported isolated candidate gene associations without independent replication, consistent with higher false-positive risk. Collectively, these findings suggest that replication failure reflects both methodological limitations and biological complexity, including heterogeneous CIPN phenotyping, ancestry differences, treatment variation, and modest true effect sizes. Future progress will require harmonised phenotyping, multi-ancestry cohorts, standardised reporting, and polygenic or pathway-level approaches rather than reliance on single SNP.

**TABLE 9:** Replication patterns stratified by methodological quality (Q-Genie score) across 72 included studies.

Q-genie category	Score range	No. studies	% of total	Predominant study designs	Common findings
High quality	≥60	32	52.4%	GWAS, prospective cohorts, larger multicentre validation studies	Included most robust positive signals such as ***CEP72*** and ***ETAA1*** (vincristine), ***EPHA5*** *,* ***FGD4*** *,* ***FZD3*** *,* ***SCN9A*** *,* ***S1PR1*** (taxanes), and recurrent ***GSTP1/ABCC2*** associations (platinum agents). However, several higher-quality studies also reported null replication, attenuated effect sizes, or discordant directionality, particularly for ***ABCB1*** *,* ***CYP2C8*** *,* ***CYP3A5*** *,* ***GSTP1***, and multiple GWAS loci that failed independent validation
Moderate quality	45–59	23	37.7%	Mixed candidate gene and cohort studies	Both positive and null findings were common. Reported associations included ***ABCB1*** *,* ***CYP1B1*** *,* ***ARHGEF10*** *,* ***FGD4*** *,* ***TRPV1*** *,* ***MTHFR*** *,* ***EDN1*** *,* and ***IL17RD***, but many were observed in single cohorts only. Several studies found previously proposed loci to be non-significant, weaker than earlier reports, or significant only under selected models/subgroup analyses. External replication was uncommon
Lower quality	<45	6	9.9%	Small candidate gene studies, retrospective analyses	Positive findings were often isolated and based on limited sample sizes, including single-study signals in metabolism, transporter, or inflammatory genes. Null findings were less frequently emphasised, and many reported associations lacked replication. Results were often sensitive to model choice, multiple-testing thresholds, or sparse event numbers

### 4.2 Biological convergence despite genetic heterogeneity

Despite inconsistent replication at the level of individual variants, a clear pattern of biological convergence emerges across studies. Genetic associations consistently implicate pathways related to neuronal structure and function, drug metabolism, and cellular stress responses.

Vincristine-induced peripheral neuropathy (VIPN), particularly relevant in pediatric acute lymphoblastic leukaemia (ALL), remains the most extensively studied phenotype. The most consistently replicated associations were found in *CEP72* and *ETAA1*, genes involved in microtubule formation and DNA damage-response pathways, respectively, both central to neuronal susceptibility (Diouf et al., 2015; Van De Velde et al., 2022). Additional candidate genes such as *NDRG1, GARS,* and *FGD4*, implicated in axonal growth and cytoskeletal maintenance, support a mechanistic model in which impaired neuronal structural integrity contributes to VIPN risk (Van De Velde et al., 2022). MicroRNA polymorphisms (e.g., *miR-3117, miR-4481*) may act as upstream regulators of neuroplasticity-related targets, though require further validation (Gutierrez-Camino et al., 2018). Within the manually curated dataset, fewer loci were reported for vincristine, with the most consistent evidence again observed for variants in *CEP72* (Klumpers et al., 2022; Mahmoudpour et al., 2018; Wright et al., 2019). However, direct comparison revealed substantial heterogeneity, with rs924607 and rs868649 showing null or inconsistent associations after covariate adjustment in curated analyses.

For taxane-induced peripheral neuropathy (TIPN), replicated associations were observed in *EPHA5, EPHA6,* and *FZD3*, all involved in axon guidance and neurite development, contrasting with variants in *SCN9A*, which relate to altered ion channel excitability (Baldwin et al., 2012; Boora et al., 2015; Chen et al., 2020; Leandro-García et al., 2013; Tanabe et al., 2020). The repeated identification of *FGD4* across both vincristine and taxane cohorts suggests overlapping microtubule-dependent mechanisms of axonal maintenance (Baldwin et al., 2012; Van De Velde et al., 2022). Variants in pharmacokinetic genes such as *CYP2C8, CYP3A5* and *ABCB1* influence drug metabolism and efflux, although their effects differ across populations and treatment protocols, emphasizing the need to integrate pharmacokinetic and neuronal susceptibility factors in predictive models (Abraham et al., 2014; Boora et al., 2015; Leskelä et al., 2011; Rizzo et al., 2010; Vargas-Aliaga et al., 2024). Within the manually curated set, TIPN showed the strongest and most numerous genetic associations, including genome-wide significant loci and variants mapping to genes involved in neuronal structure, cytoskeletal regulation, transcriptional control, and immune signalling (Adjei et al., 2021; Chua et al., 2020; Mahmoudpour et al., 2018). Notably, the association of rs74497159 in *S1PR1* highlights sphingosine-1-phosphate signalling as a biologically plausible and potentially druggable pathway in paclitaxel-induced CIPN (Chua et al., 2020). However, replicated signals in *EPHA5, FZD3*, and *FGD4* were not uniformly supported, with several variants showing weaker effects, loss of significance after correction, or variable effect direction. In contrast, *S1PR1* was largely absent from earlier studies identified through database and register search.

Evidence for platinum-induced peripheral neuropathy (PIPN) is derived predominantly from candidate gene studies. Recurrent associations with *ABCC1, ABCC2, ABCC4*, and *GSTP1* highlight impaired detoxification and reduced efflux of platinum compounds in the context of neuronal oxidative stress (Cecchin et al., 2013; Chen et al., 2020; Hong et al., 2011; Johnson et al., 2015; Katayanagi et al., 2019; Kumamoto et al., 2013; Lecomte et al., 2006). Variants in DNA repair genes, including *ERCC1, RAD51, MSH3,* and *MGMT,* modulate risk, consistent with the neurotoxic effects of unrepaired DNA crosslinks (Chen et al., 2010; Johnson et al., 2015). The recently identified *TRPV1* rs879207 variant suggests a mechanistic link between nociceptive signalling and platinum-induced hypersensitivity, though functional studies are needed (De Jong et al., 2023). Within the curated dataset, oxaliplatin studies identified multiple loci linked to neuropathy severity and recovery, implicating synaptic, calcium, and extracellular matrix–related pathways (Klumpers et al., 2022; Mahmoudpour et al., 2018; Wright et al., 2019). However, variants in *GSTP1, ABCC2*, and DNA repair genes (*ERCC1, XRCC1*) demonstrated marked variability across both data sets, with effect directions ranging from protective to deleterious.

For bortezomib-induced peripheral neuropathy (BIPN), evidence remains comparatively limited. Although several GWAS have reported suggestive loci (e.g., *PKNOX1, PRDM6, DCC*), none has achieved genome-wide significance or been independently validated (Campo et al., 2018; García-Sanz et al., 2017; Magrangeas et al., 2016). Candidate gene studies more consistently implicate *MTHFR* (rs1801131), *EDN1* (rs5370), *IL17RD* (rs1545981) and *ABCC6* (rs8058696), pointing to roles in folate metabolism, vascular regulation, inflammatory signalling and detoxification (Broyl et al., 2010; Campo et al., 2017; Zhang et al., 2024). Inconsistent effect directions may reflect ancestry-related allele frequency differences and phenotype heterogeneity. Notably, the repeated involvement of ABCC family genes across platinum and bortezomib studies suggests a shared detoxification-related pathway contributing to neurotoxicity (Campo et al., 2018; Zhang et al., 2024).

Across all drug classes, the most replicated loci, including *CEP72, ETAA1, EPHA5, FGD4* and *ABCC2*, converge on pathways related to microtubule integrity, axon guidance, DNA repair and drug efflux. Together, these genes support a polygenic model of CIPN characterized by axonal degeneration driven by oxidative stress, disrupted calcium homeostasis and impaired cytoskeletal stability (Starobova and Vetter, 2017).

Importantly, while individual variants often show inconsistent replication or opposing effects across studies, the recurrence of these biological pathways suggests that CIPN susceptibility reflects a polygenic architecture with pathway-level convergence. This indicates that focusing on individual SNPs may be insufficient to capture the complexity of CIPN risk, and that pathway-based or systems-level approaches may provide greater insight. Although polygenic risk scores (PRS) have been explored in recent work (Hooshmand et al., 2022), they remain uncommon. PRS approaches may eventually provide valuable insights into cumulative genetic risk across drug classes, provided they are developed using harmonized, multi-ancestry datasets.

### 4.3 Implications for pharmacogenomic biomarker development

A key implication of these findings is that no genetic variants currently meet criteria for clinical implementation in CIPN risk prediction. Despite extensive investigation, the lack of robust, reproducible associations limits the utility of pharmacogenomic testing in guiding treatment decisions or identifying high-risk patients.

To overcome these limitations, future studies must adopt more rigorous and standardised approaches. Harmonisation of phenotyping is essential, including consistent use of validated neuropathy assessment tools and clear definitions of clinical endpoints. Large, well-powered multi-ancestry GWAS are required to improve reproducibility and identify variants with consistent effects across populations.

In addition, standardised reporting practices, including clear annotation of effect alleles and statistical thresholds, are critical to enable cross-study comparisons and meta-analysis. Functional validation of candidate variants is also needed to establish biological relevance and distinguish true causal relationships from statistical associations.

Finally, integration of genetic data with other molecular and clinical datasets through multi-omics approaches offers a promising strategy to improve predictive accuracy. Combining genomic, transcriptomic, proteomic, and clinical data may enable the development of more robust models for risk stratification and precision oncology in CIPN.

Importantly, even if all reported risk alleles are true, their small effect sizes explain only a limited proportion of CIPN risk, with known loci likely accounting for no more than ∼5–15% of variance, making genetics alone insufficient for clinical prediction. This level of variance is inadequate for high-stakes decisions such as dose reduction but may be useful for lower-risk applications like trial enrichment or targeted monitoring. Overall, these findings support a highly polygenic, multifactorial CIPN architecture requiring integrated models combining genetics with clinical, pharmacokinetic and biological factors (Hooshmand et al., 2022; Starobova and Vetter, 2017).

### 4.4 Conclusions

In summary, this systematic review demonstrates that while numerous genetic associations with CIPN have been reported, the field is characterised by poor replication, methodological heterogeneity, and limited translational progress. However, the convergence of findings across key biological pathways supports a polygenic and mechanistically coherent model of CIPN susceptibility. Advancing pharmacogenomics in this field will require coordinated efforts to standardise study design, expand cohort diversity, and integrate functional and multi-omics data to enable clinically actionable precision medicine approaches.

## Data Availability

The original contributions presented in the study are included in the article/Supplementary Material, further inquiries can be directed to the corresponding authors.
