# Supplementary material for: Why pharmacogenomic biomarkers for chemotherapy-induced peripheral neuropathy fail: a systematic review of genetic associations, replication, and clinical translation
Source: Front Pharmacol. 2026 Jul 9;17:1842379. doi: 10.3389/fphar.2026.1842379 (PMC13391509; doi:10.3389/fphar.2026.1842379)
Supplement: Supplementary file 1 [file DataSheet1.zip › Supplementary file 1.docx]

***Table. Platinum Derivates: SNPs identified in candidate gene association studies of platinum-induced peripheral neuropathy.*** *Genetic variants associated with platinum-induced peripheral neuropathy reported exclusively in candidate gene association studies.* ***Columns:*** *rsID and gene as reported in the original publication; reported gene function obtained from NCBI’s dbSNP annotation; reference and alternate alleles based on SNP Nexus (GRCh38); effect allele and effect-allele frequency as stated by the authors (NR = not reported); sample size, unadjusted and adjusted p-values, and covariates used for adjustment as specified in the source study; effect size (default = odds ratio, unless otherwise indicated); 95% confidence interval; and study details, including citation and study type.*

| **rsID** | **Gene** | **Reported gene function** | **Reference allele** | **Alternate allele** | **Effect allele** | **Effect allele frequency** | **Sample size** | **Unadjusted P- value** | **Adjusted P-value** | **Outcomes adjusted for** | **Effect size** | **Confidence interval** | **Study name** | **Study type** | **CIPN trait studied** |
| --- | --- | --- | --- | --- | --- | --- | --- | --- | --- | --- | --- | --- | --- | --- | --- |
| rs1799811 | *GSTP1* | Function in xenobiotic metabolism and play a role in susceptibility to cancer, and other diseases |  |  | G | 0.098 | 125 |  | 0.032 |  | NR | NR | [59] | Prospective observational pharmacogenetic study | Severity |
| rs879207 | *TRPV1* | Protein encoded by this gene is a receptor for capsaicin and is a non-selective cation channel. | A | G | G |  | 320 |  | 0.012 |  | 5.2 | 2.1–12.8 | [37] | Candidate gene association - Prospective | Severity |
| rs3753753 | *GPX7* | Enables catalase activity. Predicted to be involved in cellular response to oxidative stress. Implicated in Barrett's adenocarcinoma and Barrett's esophagus | C | G or T | CC (haplotype) |  | 950 | 0.0015 | 0.0028 |  | 1.7 | 1.20–2.40 | [54] | Candidate gene association - Observational, Prospective cohort | Severity |
| rs1729786 | *ABCC4* | Member of the superfamily of ATP-binding cassette (ABC) transporters. Plays a role in cellular detoxification as a pump for its substrate, organic anions. It may also function in prostaglandin-mediated cAMP signaling in ciliogenesis |  |  | NA |  | 950 | 0.0377 | 0.006 |  | 0.63 | 0.45–0.87 |  |  | Severity |
| rs11016884 | *MGMT* | Protein encoded by this gene is a DNA repair protein that is involved in cellular defense against mutagenesis and toxicity from alkylating agents | T | C | NA |  | 950 | 0.0556 | 0.0104 |  | 0.64 | 0.46–0.90 |  |  | Severity |
| rs3092981 | *RAD51* | Known to be involved in the homologous recombination and repair of DNA. This protein can interact with the ssDNA-binding protein RPA and RAD52, and it is thought to play roles in homologous pairing and strand transfer of DNA | C | A or T | NA |  | 950 | 0.1457 | 0.0445 |  | 0.59 | 0.35–0.99 |  |  | Severity |
| rs26279 | *MSH3* | Protein encoded by this gene forms a heterodimer with MSH2 to form MutS beta, part of the post-replicative DNA mismatch repair system | G | A | NA |  |  | 0.3957 | 0.0467 |  | 1.39 | 1.01–1.93 |  |  | Severity |
| rs6492763 | *ABCC4* | Member of the superfamily of ATP-binding cassette (ABC) transporters. Plays a role in cellular detoxification as a pump for its substrate, organic anions. It may also function in prostaglandin-mediated cAMP signaling in ciliogenesis |  |  | NA |  | 950 | 0.1599 | 0.0406 |  | 1.42 | 1.02–1.98 |  |  | Severity |
| rs1695 | *GSTP1* | Function in xenobiotic metabolism and play a role in susceptibility to cancer, and other diseases | A | G | G (Val) |  | 52 | 0.03 | NA | Tumor diff., CEA, ECOG | HR 0.47 | 0.21–1.04 | [52] | Candidate Gene sub-study within a clinical trial | Severity |
| rs1695 | *Ile105Val of GSTP1* |  |  |  |  |  | 52 | 0.03 | 0.06 | Tumor differentiation, baseline CEA | 0.47 | .23-.97 |  |  |  |
| rs1695 | *GSTP1* |  |  |  | Ile (A) |  | 64 | 0.02 | 0.03 | Age, gender, regimen | OR 5.75, 5.54 adj | 1.08–30.74 | [70] | Candidate Gene Association Study (Prospective cohort) | Severity |
| rs2230641 | *CCNH* | Gene belongs to the highly conserved cyclin family. Participate in two different transcriptional regulation processes, suggesting an important link between basal transcription control and the cell cycle machinery | A | G | C |  | 413 | 0.042 | 0.014 | Age, gender, regimen, dose | OR 5.03 C/C vs T | 1.19–5.07 | [32] | Candidate gene association - Observational, Prospective Cohort | onset |
| rs843748 | *ACYP2, TSPYL6* | Can hydrolyze the phosphoenzyme intermediate of different membrane pumps, particularly the Ca2+/Mg2+-ATPase from sarcoplasmic reticulum of skeletal muscle. Gene encodes the muscle-type isoform (MT) | G | A | A | 38% | 150 | 0.028 | 0.008 |  | .45/.27 | (0.22–0.94), (0.10–0.75) | [99] | Retrospective validation (of GWAS findings) cohort with meta analysis | Severity |
| rs1695 | *GSTP1* | Function in xenobiotic metabolism and play a role in susceptibility to cancer, and other diseases |  |  | G (Val) | 0.247 | 166 | 0.012 |  |  | 3.8 | 1.3–11.2 | [24] | Prospective observational pharmacogenetic study | Severity |
| rs11615 | *ERCC1-118* |  |  |  | T | 0.376 | 166 | 0.047 |  |  | 2.4 | 1.1–5.4 |  |  | Severity |
| rs13181 | *XPD-751* |  |  |  | C | 0.229 | 166 | 0.041 |  |  | 2.2 | 1.0–4.9 |  |  | Severity |
| rs1695 | *GSTP1* | Function in xenobiotic metabolism and play a role in susceptibility to cancer, and other diseases | A | G | G |  | 63 | 0.05 | 0.03 | Multivariate logistic regression | 6.08 | 1.15–32.18 | [64] | Pharmacogenetic - Observational, Prospective Cohort | Severity |
| rs2074087 | *ABCC1* | Member of the superfamily of ATP-binding cassette (ABC) transporters. Protein functions as a multispecific organic anion transporter, with oxidized glutatione, cysteinyl leukotrienes, and activated aflatoxin B1 as substrates |  |  | C | 0.08 | 144 |  | 0.017 | Age, sex, neoadj. tx, oxaliplatin dose | OR 0.43 (0.22–0.86) | 0.22–0.86 | [20] | Prospective validation pharmacogenomic study | Severity |
| rs35587 | *ABCC1* |  |  |  | C | 0.2455 | 144 |  | 0.0375 | Age, sex, neoadj. tx, oxaliplatin dose | 0.47 | .23-.96 |  |  | Severity |
| rs1885301 | *ABCC2* | ABC proteins transport various molecules across extra- and intra-cellular membranes. This protein is expressed in the canalicular (apical) part of the hepatocyte and functions in biliary transport. Substrates include anticancer drugs such as vinblastine; therefore, this protein appears to contribute to drug resistance in mammalian cells |  |  | A | 0.5982 | 144 |  | 0.0072 | Age, sex, neoadj. tx, oxaliplatin dose | 3.06 | 1.35-6.92 |  |  | Severity |
| rs717620 | *ABCC2* |  |  |  | T | 0.2455 | 144 |  | 0.0164 | Age, sex, neoadj. tx, oxaliplatin dose | 14.39 | 1.63-127.02 |  |  | Severity |
| rs2273697 | *ABCC2* |  |  |  | A | 0.134 | 144 |  | 0.00434 | Age, sex, neoadj. tx, oxaliplatin dose | 0.44 | .20-.98 |  |  | Severity |
| rs4148396 | *ABCC2* |  |  |  | T | 0.4629 | 144 |  | 0.0048 | Age, sex, neoadj. tx, oxaliplatin dose | 4.69 | 1.6-13.74 |  |  | Severity |
| rs3740066 | *ABCC2* |  |  |  | T | 0.4545 | 144 |  | 0.0231 | Age, sex, neoadj. tx, oxaliplatin dose | 2.99 | 1.16-7.70 |  |  | Severity |
| rs2622604 | *ABCG2* |  |  |  | T | 0.3092 | 144 |  | 0.0478 | Age, sex, neoadj. tx, oxaliplatin dose | 3.61 | 1.01-12.88 |  |  | Severity |
| rs10486003 | *TAC1* | Function as neurotransmitters which interact with nerve receptors and smooth muscle cells | C | T |  |  | 343 | 4.84 × 10⁻⁷ |  |  | 0.32 | 0.19–0.52 | [104] | Prospective and validation cohort | Chronic, severe (≥2/3) |
| rs2338 | *FOXC1, GMDS* | Specific function of this gene has not yet been determined, thought to play a role in the regulation of embryonic and ocular development | G | A |  |  | 343 | 4.63 × 10⁻⁶ |  |  | 2.27 | 1.58-3.26 |  |  | Chronic, severe (≥2/3) |
| rs830884 | *ITGA1, PELO* |  | C | T |  |  | 343 | 1.74 × 10⁻⁶ |  |  | 0.32 | .19-.54 |  |  | Chronic, severe (≥2/3) |
| rs 843748 | *ACYP2* | Can hydrolyze the phosphoenzyme intermediate of different membrane pumps, particularly the Ca2+/Mg2+-ATPase from sarcoplasmic reticulum of skeletal muscle. Gene encodes the muscle-type isoform (MT) | G | A |  |  | 343 | 1.01 × 10⁻⁵ |  |  | 2.43 | 1.61-3.68 |  |  | Chronic, severe (≥2/3) |
| rs797519 | *DLEU7* |  | G | C |  |  | 343 | 8.21 × 10⁻⁵ |  |  | 0.5 | .35-.72 |  |  | Chronic, severe (≥2/3) |
| rs4936453 | *BTG4, POU2AF1* | Member of the BTG/Tob family. Has structurally related proteins that appear to have antiproliferative properties | T | G |  |  | 343 | 9.86 × 10⁻⁵ |  |  | 0.49 | .33-.71 |  |  | Chronic, severe (≥2/3) |
| rs12023000 | *CAMK2N1* | Predicted to be located in neuronal cell body and synapse. Implicated in ovarian cancer; ovarian carcinoma; and prostate adenocarcinoma | A | G |  |  | 343 | 8.81 × 10⁻⁵ |  |  | 0.47 | .31-.70 |  |  | Chronic, severe (≥2/3) |
| rs17140129 | *FARS2, LYRM4* | Encodes a protein that transfers phenylalanine to its cognate tRNA. Protein localizes to the mitochondrion and plays a role in mitochondrial protein translation | A | G |  |  | 343 | 3.23 × 10⁻⁵ |  |  | 3.34 | 1.81-6.35 |  |  | Chronic, severe (≥2/3) |
| rs6924717 | *FARS2, LYRM4* |  |  |  |  |  | 343 | 3.23 × 10⁻⁵ |  |  | 3.34 | 1.81-6.35 |  |  | Chronic, severe (≥2/3) |
