# Supplementary material for: Why pharmacogenomic biomarkers for chemotherapy-induced peripheral neuropathy fail: a systematic review of genetic associations, replication, and clinical translation
Source: Front Pharmacol. 2026 Jul 9;17:1842379. doi: 10.3389/fphar.2026.1842379 (PMC13391509; doi:10.3389/fphar.2026.1842379)
Supplement: Supplementary file 1 [file DataSheet1.zip › Supplementary Material 1,2.PDF]

Supplementary Materials for

Why Pharmacogenomic Biomarkers for Chemotherapy-Induced Peripheral Neuropathy Fail: A Systematic Review of Genetic Associations, Replication, and Clinical Translation

Aditya Maganti[1], Ishara Rankothge[1], Hunter Sakadales[1], Ethan Kok[2], James Britton[1], Neil Rao[1], Svetlana Shaunova[3], Katherine Shimel[3], Paula Manuela Zambrano Rojas[3], Susanna B. Park[4], Daniel Schweitzer[5], E-Liisa Lakso[6], Loic Yengo[3], Irina Vetter[3, 7*] and Hana Starobova[3*]

* Corresponding authors

[1] Medical School, The University of Queensland, St. Lucia, QLD, Australia
[2] School of the Environment (Biological Sciences), The University of Queensland, St Lucia, QLD, Australia
[3] Institute for Molecular Bioscience, The University of Queensland, St Lucia, QLD, Australia
[4]School of Medical Sciences, Faculty of Medicine and Health, The University of Sydney, Sydney, Australia
[5]Mater Centre of Neuroscience, Mater Hospital, Wesley Hospital, University of Queensland
[6] Mater Research Institute-University of Queensland, South Brisbane, QLD, Australia
[7]The School of Pharmacy and Pharmaceutical Sciences, The University of Queensland, Woolloongabba, QLD, Australia

Supplementary Materials 1:

General search structure:

(Risk factors)

AND

(Chemotherapy terms)
AND

(Gene terms)

AND

(Peripheral neuropathy section)

---

1. **PubMed:**

("Risk Factors"[Mesh] OR factor*[tiab] OR determinant*[tiab])

AND

(chemotherap*[tiab] OR "antibody drug conjugate*"[tiab] OR "Antineoplastic Agents"[Mesh])

AND

("genome-wide association study"[MeSH] OR "polymorphism, single nucleotide"[MeSH] "gene expression"[tiab] OR "genetic association studies"[MeSH] OR "genetic predisposition"[tiab] OR "genetic risk"[tiab] OR "genetic variability"[tiab] OR genetic*[tiab] OR geno*[tiab] OR SNP[tiab] OR pharmacogen*[tiab] OR polymorphism*[tiab] OR GWAS[tiab] OR "candidate gene"[tiab] OR "association study"[tiab] OR "genomics"[tiab])

AND

("Peripheral Neuropath*"[tiab] OR "peripheral neurotox*"[tiab] OR "chemotherapy induced pain"[tiab])

NOT (animals [mh] NOT humans [mh])

NOT ("clinical trial protocol"[pt] OR "clinical conference"[pt] OR congress[pt] OR "case reports"[pt] OR "systematic review"[pt])

---

2. **Embase:**

('risk factors'/exp OR factor*:ti,ab OR determinant*:ti,ab)

AND

('genome-wide association study'/exp OR 'single nucleotide polymorphism'/exp OR 'gene expression':ti,ab OR 'genetic predisposition':ti,ab OR 'genetic risk':ti,ab OR 'genetic variability':ti,ab OR 'genetic*':ti,ab OR 'genom*':ti,ab OR 'polymorphism*':ti,ab OR gwas:ti,ab OR 'candidate gene':ti,ab OR 'association study':ti,ab OR 'genomics':ti,ab)

AND

('chemotherapy'/exp OR 'antibody conjugate*':ti,ab OR 'antineoplastic agent'/exp)

AND

('peripheral neuropathy'/exp OR 'peripheral neurotox*':ti,ab OR 'chemotherapy induced pain':ti,ab)

NOT ('conference abstract'/it OR 'review'/it OR 'systematic review'/it)

---

3. Cochrane Library:

([mh "Risk Factors"] OR factor*:ti,ab OR determinant*:ti,ab)

AND

(chemotherap*:ti,ab OR ("antibody drug" NEXT conjugate*):ti,ab OR [mh "Antineoplastic Agents"])

AND

([mh "genome-wide association study"] OR [mh "polymorphism, single nucleotide"] OR [mh "genetic association studies"] OR "genetic predisposition":ti,ab OR "genetic risk":ti,ab OR "genetic variability":ti,ab OR genetic*:ti,ab OR geno*:ti,ab OR SNP:ti,ab OR pharmacogen*:ti,ab OR polymorphism*:ti,ab OR GWAS:ti,ab OR "candidate gene":ti,ab OR "association study":ti,ab OR genomics:ti,ab)

AND

(("Peripheral" NEXT Neuropath*):ti,ab OR ("peripheral" NEXT neurotox*):ti,ab OR "chemotherapy induced pain":ti,ab)

NOT ([mh animals] NOT [mh humans])

NOT ("clinical trial protocol":pt OR "clinical conference":pt OR congress:pt OR "case reports":pt OR "systematic review":pt)

---

4. Web of Science:

("Risk Factors" OR factor* OR determinant*)

AND

(chemotherap* OR "antibody drug conjugate*" OR "Antineoplastic Agents")

AND

("genome-wide association study" OR "polymorphism, single nucleotide" OR "genetic association studies" OR "genetic predisposition" OR "genetic risk" OR "genetic variability" OR genetic* OR geno* OR SNP OR pharmacogen* OR polymorphism* OR GWAS OR "candidate gene" OR "association study" OR genomics)

AND

("Peripheral Neuropath*" OR "peripheral neurotox*" OR "chemotherapy induced pain")

NOT (animals NOT humans)

NOT ("clinical trial protocol" OR "clinical conference" OR congress OR "case reports" OR "systematic review")

Supplementary Materials 2:

Studies identified via databases and registers:

Vicristine: (n = 15)

Mufti K, Cordova M, Scott EN, Trueman JN, Lovnicki JM, Loucks CM, Rassekh SR, Ross CJD, Carleton BC, On behalf of the Canadian Pharmacogenomics Network for Drug Safety C, Groeneweg GSS, Higginson M, Chang W-C, Li K, Miao F, Yau D, Pecheux L, Gyawali B, Perreault A, Abbasi F, Guilcher G, Riabowol G, Jong GT, Staub M, Cuvelier G, Felton K, Khalaj S, Rieder M, Abuzgaia A, Lewis T, Ghimire H, Nathan P, Johnston D, Ibrahim M, Bussières JF, Jean-Baptiste T-R, Lebel D, Krajinovic M, Tran TH, Goralski K, Forbrigger Z, Kulkarni K (2024) Genomic variations associated with risk and protection against vincristine-induced peripheral neuropathy in pediatric cancer patients. npj Genom Med 9: 56 doi:10.1038/s41525-024-00443-7

Yamada H, Ohmori R, Okada N, Nakamura S, Kagawa K, Fujii S, Miki H, Ishizawa K, Abe M, Sato Y (2022) A machine learning model using SNPs obtained from a genome-wide association study predicts the onset of vincristine-induced peripheral neuropathy. Pharmacogenomics J 22: 241-246 doi:10.1038/s41397-022-00282-8

Aplenc R, Glatfelter W, Han P, Rappaport E, La M, Cnaan A, Blackwood MA, Lange B, Rebbeck T (2003) CYP3A genotypes and treatment response in paediatric acute lymphoblastic leukaemia. Br J Haematol 122: 240-244 doi:10.1046/j.1365-2141.2003.04430.x

Egbelakin A, Ferguson MJ, MacGill EA, Lehmann AS, Topletz AR, Quinney SK, Li L, McCammack KC, Hall SD, Renbarger JL (2011) Increased risk of vincristine neurotoxicity associated with low CYP3A5 expression genotype in children with acute lymphoblastic leukemia. Pediatric Blood & Cancer 56: 361-367 doi:10.1002/pbc.22845

Sepe DM, McWilliams T, Chen J, Kershenbaum A, Zhao H, La M, Devidas M, Lange B, Rebbeck TR, Aplenc R (2012) Germline genetic variation and treatment response on CCG-1891. Pediatric Blood & Cancer 58: 695-700 doi:10.1002/pbc.23192

Kishi S, Cheng C, French D, Pei D, Das S, Cook EH, Hijiya N, Rizzari C, Rosner GL, Frudakis T, Pui C-H, Evans WE, Relling MV (2007) Ancestry and pharmacogenetics of antileukemic drug toxicity. Blood 109: 4151-4157 doi:10.1182/blood-2006-10-054528

Diouf B, Crews KR, Lew G, Pei D, Cheng C, Bao J, Zheng JJ, Yang W, Fan Y, Wheeler HE, Wing C, Delaney SM, Komatsu M, Paugh SW, McCorkle JR, Lu X, Winick NJ, Carroll WL, Loh ML, Hunger SP, Devidas M, Pui C-H, Dolan ME, Relling MV, Evans WE (2015) Association of an Inherited Genetic Variant With Vincristine-Related Peripheral Neuropathy in Children With Acute Lymphoblastic Leukemia. JAMA 313: 815 doi:10.1001/jama.2015.0894

Van De Velde ME, Uittenboogaard A, Yang W, Bonten E, Cheng C, Pei D, Van Den Berg MH, Van Der Sluis IM, Van Den Bos C, Abbink FCH, Van Den Heuvel-Eibrink MM, Segers H, Chantrain C, Van Der Werff Ten Bosch J, Willems L, Evans WE, Kaspers GJL (2022) Genetic Polymorphisms Associated with

Vincristine Pharmacokinetics and Vincristine-Induced Peripheral Neuropathy in Pediatric Oncology Patients. Cancers 14: 3510 doi:10.3390/cancers14143510

Gutierrez-Camino Á, Umerez M, Martin-Guerrero I, García De Andoin N, Santos B, Sastre A, Echebarria-Barona A, Astigarraga I, Navajas A, Garcia-Orad A (2018) Mir-pharmacogenetics of Vincristine and peripheral neurotoxicity in childhood B-cell acute lymphoblastic leukemia. Pharmacogenomics J 18: 704-712 doi:10.1038/s41397-017-0003-3

Christofyllakis K, Kaddu-Mulindwa D, Lesan V, Rixecker T, Kos IA, Held G, Regitz E, Pfreundschuh M, Bittenbring JT, Thurner L, Poeschel V, Ziepert M, Altmann B, Bewarder M (2024) An inherited genetic variant of the CEP72 gene is associated with the development of vincristine-induced peripheral neuropathy in female patients with aggressive B-cell lymphoma. Ann Hematol 103: 4599-4606 doi:10.1007/s00277-024-05973-9

Yuan Y, Hu W, Chen C, Yao R, Zhang S, Zhu X, Xu B, Huang Z, Zhang S, Wang X, Zheng M, Huang X, Standing JF (2025) Pharmacokinetic, Pharmacodynamic and Pharmacogenetic Studies Related to Vincristine-Induced Peripheral Neuropathy in Chinese Pediatric ALL Patients. Clin Pharma and Therapeutics 117: 454-464 doi:10.1002/cpt.3462

Guilhaumou R, Solas C, Bourgarel-Rey V, Quaranta S, Rome A, Simon N, Lacarelle B, Andre N (2011) Impact of plasma and intracellular exposure and CYP3A4, CYP3A5, and ABCB1 genetic polymorphisms on vincristine-induced neurotoxicity. Cancer Chemother Pharmacol 68: 1633-1638 doi:10.1007/s00280-011-1745-2

Moore AS, Norris R, Price G, Nguyen T, Ni M, George R, Van Breda K, Duley J, Charles B, Pinkerton R (2011) Vincristine pharmacodynamics and pharmacogenetics in children with cancer: A limited-sampling, population modelling approach. J Paediatrics Child Health 47: 875-882 doi:10.1111/j.1440-1754.2011.02103.x

Cho H-J, Eom H-S, Kim H-J, Kim I-S, Lee GW, Kong S-Y (2010) Glutathione-S-transferase genotypes influence the risk of chemotherapy-related toxicities and prognosis in Korean patients with diffuse large B-cell lymphoma. Cancer Genetics and Cytogenetics 198: 40-46 doi:10.1016/j.cancergencyto.2009.12.004

Broyl A, Corthals SL, Jongen JL, Van Der Holt B, Kuiper R, De Knegt Y, Van Duin M, El Jarari L, Bertsch U, Lokhorst HM, Durie BG, Goldschmidt H, Sonneveld P (2010) Mechanisms of peripheral neuropathy associated with bortezomib and vincristine in patients with newly diagnosed multiple myeloma: a prospective analysis of data from the HOVON-65/GMMG-HD4 trial. The Lancet Oncology 11: 1057-1065 doi:10.1016/S1470-2045(10)70206-0

Taxanes: (n = 24)

Baldwin RM, Owzar K, Zembutsu H, Chhibber A, Kubo M, Jiang C, Watson D, Eclov RJ, Mefford J, McLeod HL, Friedman PN, Hudis CA, Winer EP, Jorgenson EM, Witte JS, Shulman LN, Nakamura Y, Ratain MJ, Kroetz DL (2012) A Genome-Wide Association Study Identifies Novel Loci for Paclitaxel-

Induced Sensory Peripheral Neuropathy in CALGB 40101. Clinical Cancer Research 18: 5099-5109 doi:10.1158/1078-0432.CCR-12-1590

Sucheston-Campbell LE, Clay-Gilmour AI, Barlow WE, Budd GT, Stram DO, Haiman CA, Sheng X, Yan L, Zirpoli G, Yao S, Jiang C, Owzar K, Hershman D, Albain KS, Hayes DF, Moore HC, Hobday TJ, Stewart JA, Rizvi A, Isaacs C, Salim M, Gralow JR, Hortobagyi GN, Livingston RB, Kroetz DL, Ambrosone CB (2018) Genome-wide meta-analyses identifies novel taxane-induced peripheral neuropathy-associated loci. Pharmacogenetics and Genomics 28: 49-55 doi:10.1097/FPC.0000000000000318

Hooshmand K, Goldstein D, Timmins HC, Li T, Harrison M, Friedlander ML, Lewis CR, Lees JG, Moalem-Taylor G, Guennewig B, Park SB, Kwok JB (2022) Polygenic risk of paclitaxel-induced peripheral neuropathy: a genome-wide association study. J Transl Med 20: 564 doi:10.1186/s12967-022-03754-4

Vargas-Aliaga A, De La Haba M, Contreras MJ, Morales Estevez C, Porras I, Cano MT, Pulido G, Gómez MA, Flores-Paco P, Juan DLH-R, Aranda E (2024) NeuroPredict: study of the predictive value of ABCB1 genetic polymorphisms and associated clinical factors in chronic chemotherapy-induced peripheral neuropathy (CIPN). Front Pharmacol 15: 1352939 doi:10.3389/fphar.2024.1352939

Tanabe Y, Shiraishi S, Hashimoto K, Ikeda K, Nishizawa D, Hasegawa J, Shimomura A, Ozaki Y, Tamura N, Yunokawa M, Yonemori K, Takano T, Kawabata H, Tamura K, Fujiwara Y, Shimizu C (2020) Taxane-induced sensory peripheral neuropathy is associated with an SCN9A single nucleotide polymorphism in Japanese patients. BMC Cancer 20: 325 doi:10.1186/s12885-020-06834-0

Bosó V, Herrero MJ, Santaballa A, Palomar L, Megias JE, De La Cueva H, Rojas L, Marqués MR, Poveda JL, Montalar J, Aliño SF (2014) Snps and Taxane Toxicity in Breast Cancer Patients. Pharmacogenomics 15: 1845-1858 doi:10.2217/pgs.14.127

Hertz DL, Roy S, Motsinger-Reif AA, Drobish A, Clark LS, McLeod HL, Carey LA, Dees EC (2013) CYP2C8*3 increases risk of neuropathy in breast cancer patients treated with paclitaxel. Annals of Oncology 24: 1472-1478 doi:10.1093/annonc/mdt018

Sissung TM, Mross K, Steinberg SM, Behringer D, Figg WD, Sparreboom A, Mielke S (2006) Association of ABCB1 genotypes with paclitaxel-mediated peripheral neuropathy and neutropenia. European Journal of Cancer 42: 2893-2896 doi:10.1016/j.ejca.2006.06.017

De Graan A-JM, Elens L, Sprowl JA, Sparreboom A, Friberg LE, Van Der Holt B, De Raaf PJ, De Bruijn P, Engels FK, Eskens FALM, Wiemer EAC, Verweij J, Mathijssen RHJ, Van Schaik RHN (2013) <i>CYP3A4*22</i> Genotype and Systemic Exposure Affect Paclitaxel-Induced Neurotoxicity. Clinical Cancer Research 19: 3316-3324 doi:10.1158/1078-0432.CCR-12-3786

Sucheston LE, Zhao H, Yao S, Zirpoli G, Liu S, Barlow WE, Moore HCF, Thomas Budd G, Hershman DL, Davis W, Ciupak GL, Stewart JA, Isaacs C, Hobday TJ, Salim M, Hortobagyi GN, Gralow JR, Livingston RB, Albain KS, Hayes DF, Ambrosone CB (2011) Genetic predictors of taxane-induced

neurotoxicity in a SWOG phase III intergroup adjuvant breast cancer treatment trial (S0221). Breast Cancer Res Treat 130: 993-1002 doi:10.1007/s10549-011-1671-3

Marsh S, Paul J, King CR, Gifford G, McLeod HL, Brown R (2007) Pharmacogenetic Assessment of Toxicity and Outcome After Platinum Plus Taxane Chemotherapy in Ovarian Cancer: The Scottish Randomised Trial in Ovarian Cancer. JCO 25: 4528-4535 doi:10.1200/JCO.2006.10.4752

Abraham JE, Guo Q, Dorling L, Tyrer J, Ingle S, Hardy R, Vallier A-L, Hiller L, Burns R, Jones L, Bowden SJ, Dunn JA, Poole CJ, Caldas C, Pharoah PPD, Earl HM (2014) Replication of Genetic Polymorphisms Reported to Be Associated with Taxane-Related Sensory Neuropathy in Patients with Early Breast Cancer Treated with Paclitaxel. Clinical Cancer Research 20: 2466-2475 doi:10.1158/1078-0432.CCR-13-3232

Leskelä S, Jara C, Leandro-García LJ, Martínez A, García-Donas J, Hernando S, Hurtado A, Vicario JCC, Montero-Conde C, Landa I, López-Jiménez E, Cascón A, Milne RL, Robledo M, Rodríguez-Antona C (2011) Polymorphisms in cytochromes P450 2C8 and 3A5 are associated with paclitaxel neurotoxicity. Pharmacogenomics J 11: 121-129 doi:10.1038/tpj.2010.13

Kus T, Aktas G, Kalender ME, Demiryurek AT, Ulasli M, Oztuzcu S, Sevinc A, Kul S, Camci C (2016) Polymorphism of CYP3A4 and ABCB1 genes increase the risk of neuropathy in breast cancer patients treated with paclitaxel and docetaxel. OncoTargets and therapy: 5073-5080

Deng F, Laasik M, Salminen L, Lapatto L, Huhtinen K, Li Y, Hautaniemi S, Hynninen J, Niemi M, Lehtonen R (2023) Toxicity and therapy outcome associations in <i>LIG3</i> , <i>SLCO1B3</i> , <i>ABCB1</i> , <i>OPRM1</i> and <i>GSTP1</i> in high-grade serous ovarian cancer. Basic Clin Pharma Tox 132: 517-527 doi:10.1111/bcpt.13866

Abdelfattah NM, Solayman MH, Elnahass Y, Sabri NA (2021) <i>ABCB1</i> Single Nucleotide Polymorphism Genotypes as Predictors of Paclitaxel-Induced Peripheral Neuropathy in Breast Cancer. Genetic Testing and Molecular Biomarkers 25: 471-477 doi:10.1089/gtmb.2021.0014

De Jong C, Herder GJM, Van Haarlem SWA, Van Der Meer FS, Van Lindert ASR, Ten Heuvel A, Brouwer J, Egberts TCG, Deneer VHM (2023) Association between Genetic Variants and Peripheral Neuropathy in Patients with NSCLC Treated with First-Line Platinum-Based Therapy. Genes 14: 170 doi:10.3390/genes14010170

Boora GK, Kulkarni AA, Kanwar R, Beyerlein P, Qin R, Banck MS, Ruddy KJ, Pleticha J, Lynch CA, Behrens RJ, Züchner S, Loprinzi CL, Beutler AS (2015) Association of the Charcot–Marie–Tooth disease gene ARHGEF10 with paclitaxel induced peripheral neuropathy in NCCTG N08CA (Alliance). Journal of the Neurological Sciences 357: 35-40 doi:10.1016/j.jns.2015.06.056

Nakayama H, Ishida H, Iidaka M, Kato S, Nakatani K, Nakayama A, Noguchi T, Nishihara S, Oikawa S, Usami T, Mitsui Y, Ishii Y, Toshima H, Kobayashi K, Murase R, Matsumoto N, Suzuki K, Shimada K, Yoshida H, Fujita K-I (2024) Association Between <i>ABCC2</i> −24C>T and Nab-Paclitaxel-induced

Peripheral Neuropathy in Japanese Patients With Pancreatic Cancer. Anticancer Res 44: 5023-5033 doi:10.21873/anticanres.17326

Rizzo R, Spaggiari F, Indelli M, Lelli G, Baricordi OR, Rimessi P, Ferlini A (2010) Association of CYP1B1 with hypersensitivity induced by Taxane therapy in breast cancer patients. Breast Cancer Res Treat 124: 593-598 doi:10.1007/s10549-010-1034-5

Park SB, Kwok JB, Asher R, Lee CK, Beale P, Selle F, Friedlander M (2017) Clinical and genetic predictors of paclitaxel neurotoxicity based on patient- versus clinician-reported incidence and severity of neurotoxicity in the ICON7 trial. Annals of Oncology 28: 2733-2740 doi:10.1093/annonc/mdx491

Bergmann TK, Brasch-Andersen C, Gréen H, Mirza MR, Skougaard K, Wihl J, Keldsen N, Damkier P, Peterson C, Vach W, Brøsen K (2012) Impact of <i>ABCB1</i> Variants on Neutrophil Depression: A Pharmacogenomic Study of Paclitaxel in 92 Women with Ovarian Cancer. Basic Clin Pharma Tox 110: 199-204 doi:10.1111/j.1742-7843.2011.00802.x

Chen Y, Fang F, Kidwell KM, Vangipuram K, Marcath LA, Gersch CL, Rae JM, Hayes DF, Lavoie Smith EM, Henry NL, Beutler AS, Hertz DL (2020) Genetic Variation in Charcot–Marie–Tooth Genes Contributes to Sensitivity to Paclitaxel-Induced Peripheral Neuropathy. Pharmacogenomics 21: 841-851 doi:10.2217/pgs-2020-0053

Leandro-García LJ, Inglada-Pérez L, Pita G, Hjerpe E, Leskelä S, Jara C, Mielgo X, González-Neira A, Robledo M, Åvall-Lundqvist E, Gréen H, Rodríguez-Antona C (2013) Genome-wide association study identifies ephrin type A receptors implicated in paclitaxel induced peripheral sensory neuropathy. J Med Genet 50: 599-605 doi:10.1136/jmedgenet-2012-101466

Platinum-based agents: (n = 19)

Katayanagi S, Katsumata K, Mori Y, Narahara K, Shigoka M, Matsudo T, Enomoto M, Suda T, Ishizaki T, Hisada M, Nagakawa Y, Tsuchida A (2019) GSTP1 as a potential predictive factor for adverse events associated with platinum-based antitumor agent-induced peripheral neuropathy. Oncology Letters doi:10.3892/ol.2019.9907

De Jong C, Herder GJM, Van Haarlem SWA, Van Der Meer FS, Van Lindert ASR, Ten Heuvel A, Brouwer J, Egberts TCG, Deneer VHM (2023) Association between Genetic Variants and Peripheral Neuropathy in Patients with NSCLC Treated with First-Line Platinum-Based Therapy. Genes 14: 170 doi:10.3390/genes14010170

Johnson C, Pankratz VS, Velazquez AI, Aakre JA, Loprinzi CL, Staff NP, Windebank AJ, Yang P (2015) Candidate pathway-based genetic association study of platinum and platinum–taxane related toxicity in a cohort of primary lung cancer patients. Journal of the Neurological Sciences 349: 124-128 doi:10.1016/j.jns.2014.12.041

Hong J, Han S-W, Ham HS, Kim T-Y, Choi IS, Kim B-S, Oh D-Y, Im S-A, Kang GH, Bang Y-J, Kim T-Y (2011) Phase II study of biweekly S-1 and oxaliplatin combination chemotherapy in metastatic colorectal

cancer and pharmacogenetic analysis. Cancer Chemother Pharmacol 67: 1323-1331 doi:10.1007/s00280-010-1425-7

Lecomte T, Landi B, Beaune P, Laurent-Puig P, Loriot M-A (2006) Glutathione <i>S</i> -Transferase P1 Polymorphism (Ile105Val) Predicts Cumulative Neuropathy in Patients Receiving Oxaliplatin-Based Chemotherapy. Clinical Cancer Research 12: 3050-3056 doi:10.1158/1078-0432.CCR-05-2076

Custodio A, Moreno-Rubio J, Aparicio J, Gallego-Plazas J, Yaya R, Maurel J, Higuera O, Burgos E, Ramos D, Calatrava A, Andrada E, López R, Moreno V, Madero R, Cejas P, Feliu J (2014) Pharmacogenetic predictors of severe peripheral neuropathy in colon cancer patients treated with oxaliplatin-based adjuvant chemotherapy: a GEMCAD group study. Annals of Oncology 25: 398-403 doi:10.1093/annonc/mdt546

Terrazzino S, Argyriou AA, Cargnin S, Antonacopoulou AG, Briani C, Bruna J, Velasco R, Alberti P, Campagnolo M, Lonardi S, Cortinovis D, Cazzaniga M, Santos C, Kalofonos HP, Canonico PL, Genazzani AA, Cavaletti G (2015) Genetic determinants of chronic oxaliplatin-induced peripheral neurotoxicity: a genome-wide study replication and meta-analysis. J Peripheral Nervous Sys 20: 15-23 doi:10.1111/jns.12110

Chen YC, Tzeng CH, Chen PM, Lin JK, Lin TC, Chen WS, Jiang JK, Wang HS, Wang WS (2010) Influence of GSTP1 I105V polymorphism on cumulative neuropathy and outcome of FOLFOX-4 treatment in Asian patients with colorectal carcinoma. Cancer Science 101: 530-535

Kumamoto K, Ishibashi K, Okada N, Tajima Y, Kuwabara K, Kumagai Y, Baba H, Haga N, Ishida H (2013) Polymorphisms of GSTP1, ERCC2 and TS-3′UTR are associated with the clinical outcome of mFOLFOX6 in colorectal cancer patients. Oncology Letters 6: 648-654 doi:10.3892/ol.2013.1467

Cecchin E, D'Andrea M, Lonardi S, Zanusso C, Pella N, Errante D, De Mattia E, Polesel J, Innocenti F, Toffoli G (2013) A prospective validation pharmacogenomic study in the adjuvant setting of colorectal cancer patients treated with the 5-fluorouracil/leucovorin/oxaliplatin (FOLFOX4) regimen. Pharmacogenomics J 13: 403-409 doi:10.1038/tpj.2012.31

Won HH, Lee J, Park JO, Park YS, Lim HY, Kang WK, Kim JW, Lee SY, Park SH (2012) Polymorphic markers associated with severe oxaliplatin-induced, chronic peripheral neuropathy in colon cancer patients. Cancer 118: 2828-2836 doi:10.1002/cncr.26614

Vargas-Aliaga A, De La Haba M, Contreras MJ, Morales Estevez C, Porras I, Cano MT, Pulido G, Gómez MA, Flores-Paco P, Juan DLH-R, Aranda E (2024) NeuroPredict: study of the predictive value of ABCB1 genetic polymorphisms and associated clinical factors in chronic chemotherapy-induced peripheral neuropathy (CIPN). Front Pharmacol 15: 1352939 doi:10.3389/fphar.2024.1352939

Ruzzo A, Graziano F, Galli F, Giacomini E, Floriani I, Galli F, Rulli E, Lonardi S, Ronzoni M, Massidda B, Zagonel V, Pella N, Mucciarini C, Labianca R, Ionta MT, Veltri E, Sozzi P, Barni S, Ricci V, Foltran L, Nicolini M, Biondi E, Bramati A, Turci D, Lazzarelli S, Verusio C, Bergamo F, Sobrero A, Frontini L,

Magnani M (2014) Genetic markers for toxicity of adjuvant oxaliplatin and fluoropyrimidines in the phase III TOSCA trial in high-risk colon cancer patients. Sci Rep 4: 6828 doi:10.1038/srep06828

Lai JI, Tzeng CH, Chen PM, Lin JK, Lin TC, Chen WS, Jiang JK, Wang HS, Wang WS (2009) Very low prevalence of XPD K751Q polymorphism and its association with XPD expression and outcomes of FOLFOX-4 treatment in Asian patients with colorectal carcinoma. Cancer Science 100: 1261-1266 doi:10.1111/j.1349-7006.2009.01186.x

Chua W, Goldstein D, Lee CK, Dhillon H, Michael M, Mitchell P, Clarke SJ, Iacopetta B (2009) Molecular markers of response and toxicity to FOLFOX chemotherapy in metastatic colorectal cancer. Br J Cancer 101: 998-1004 doi:10.1038/sj.bjc.6605239

Antonacopoulou AG, Argyriou AA, Scopa CD, Kottorou A, Kominea A, Peroukides S, Kalofonos HP (2010) Integrin beta-3 L33P: a new insight into the pathogenesis of chronic oxaliplatin-induced peripheral neuropathy? Euro J of Neurology 17: 963-968 doi:10.1111/j.1468-1331.2010.02966.x

Kanai M, Yoshioka A, Tanaka S, Nagayama S, Matsumoto S, Nishimura T, Niimi M, Teramukai S, Takahashi R, Mori Y, Kitano T, Ishiguro H, Yanagihara K, Chiba T, Fukushima M, Matsuda F (2010) Associations between glutathione S-transferase π Ile105Val and glyoxylate aminotransferase Pro11Leu and Ile340Met polymorphisms and early-onset oxaliplatin-induced neuropathy. Cancer Epidemiology 34: 189-193 doi:10.1016/j.canep.2010.02.008

McLeod HL, Sargent DJ, Marsh S, Green EM, King CR, Fuchs CS, Ramanathan RK, Williamson SK, Findlay BP, Thibodeau SN (2010) Pharmacogenetic predictors of adverse events and response to chemotherapy in metastatic colorectal cancer: results from North American Gastrointestinal Intergroup Trial N9741. JCO 28: 3227-3233

Kweekel DM, Gelderblom H, Antonini NF, Van Der Straaten T, Nortier JWR, Punt CJA, Guchelaar H-J (2009) Glutathione-S-transferase pi (GSTP1) codon 105 polymorphism is not associated with oxaliplatin efficacy or toxicity in advanced colorectal cancer patients. European Journal of Cancer 45: 572-578 doi:10.1016/j.ejca.2008.10.015

Bortezomib: (n = 6)

Magrangeas F, Kuiper R, Avet-Loiseau H, Gouraud W, Guérin-Charbonnel C, Ferrer L, Aussem A, Elghazel H, Suhard J, Der Sakissian H, Attal M, Munshi NC, Sonneveld P, Dumontet C, Moreau P, Van Duin M, Campion L, Minvielle S (2016) A Genome-Wide Association Study Identifies a Novel Locus for Bortezomib-Induced Peripheral Neuropathy in European Patients with Multiple Myeloma. Clinical Cancer Research 22: 4350-4355 doi:10.1158/1078-0432.CCR-15-3163

García-Sanz R, Corchete LA, Alcoceba M, Chillon MC, Jiménez C, Prieto I, García-Álvarez M, Puig N, Rapado I, Barrio S, Oriol A, Blanchard MJ, De La Rubia J, Martínez R, Lahuerta JJ, González Díaz M, Mateos MV, San Miguel JF, Martínez-López J, Sarasquete ME, On behalf of the GEMPcsg (2017) Prediction of peripheral neuropathy in multiple myeloma patients receiving bortezomib and thalidomide: a genetic study based on a single nucleotide polymorphism array. Hematological Oncology 35: 746-751 doi:10.1002/hon.2337

Campo C, Da Silva Filho MI, Weinhold N, Mahmoudpour SH, Goldschmidt H, Hemminki K, Merz M, Försti A (2018) Bortezomib-induced peripheral neuropathy: A genome-wide association study on multiple myeloma patients. Hematological Oncology 36: 232-237 doi:10.1002/hon.2391

Broyl A, Corthals SL, Jongen JL, Van Der Holt B, Kuiper R, De Knegt Y, Van Duin M, El Jarari L, Bertsch U, Lokhorst HM, Durie BG, Goldschmidt H, Sonneveld P (2010) Mechanisms of peripheral neuropathy associated with bortezomib and vincristine in patients with newly diagnosed multiple myeloma: a prospective analysis of data from the HOVON-65/GMMG-HD4 trial. The Lancet Oncology 11: 1057-1065 doi:10.1016/S1470-2045(10)70206-0

Zhang Y, Zhang H, Wang J, Wei X, Qu Y, Xu F, Zhang L (2024) A genetic variant study of bortezomib-induced peripheral neuropathy in Chinese multiple myeloma patients. OR 32: 955-963 doi:10.32604/or.2023.043922

Corthals SL, Kuiper R, Johnson DC, Sonneveld P, Hajek R, Van Der Holt B, Magrangeas F, Goldschmidt H, Morgan GJ, Avet-Loiseau H (2011) Genetic factors underlying the risk of bortezomib induced peripheral neuropathy in multiple myeloma patients. Haematologica 96: 1728-1732 doi:10.3324/haematol.2011.041434

Manually curated dataset

Chua, K.C., et al., Genomewide meta-analysis validates a role for S1PR1 in microtubule targeting agent-induced sensory peripheral neuropathy. Clinical Pharmacology & Therapeutics, 2020. 108(3): p. 625-634.

Wright, G.E., et al., Pharmacogenomics of vincristine-induced peripheral neuropathy implicates pharmacokinetic and inherited neuropathy genes. Clinical Pharmacology & Therapeutics, 2019. 105(2): p. 402-410.

Adjei, A.A., et al., Genetic predictors of chemotherapy-induced peripheral neuropathy from paclitaxel, carboplatin and oxaliplatin: NCCTG/alliance N08C1, N08CA and N08CB study. Cancers, 2021. 13(5): p. 1084.

Klumpers, M.J., et al., Contribution of common and rare genetic variants in CEP72 on vincristine-induced peripheral neuropathy in brain tumour patients. British Journal of Clinical Pharmacology, 2022. 88(7): p. 3463-3473.

Zgheib, N.K., et al., Genetic polymorphisms in candidate genes are not associated with increased vincristine-related peripheral neuropathy in Arab children treated for acute childhood leukemia: a single institution study. Pharmacogenetics and genomics, 2018. 28(8): p. 189-195.

Kanai, M., et al., Large-scale prospective genome-wide association study of oxaliplatin in stage II/III colon cancer and neuropathy. Annals of Oncology, 2021. 32(11): p. 1434-1441.

Kanai, M., et al., Large-scale prospective pharmacogenomics study of oxaliplatin-induced neuropathy in colon cancer patients enrolled in the JFMC41-1001-C2 (JOIN Trial). Annals of Oncology, 2016. 27(6): p. 1143-1148.

Min, Y.G., et al., Genetic Risk Factors for Bortezomib-induced Neuropathic Pain in an Asian Population: A Genome-wide Association Study in South Korea. The Journal of Pain, 2024. 25(9): p. 104552.

Mahmoudpour, S.H., et al., Chemotherapy-induced peripheral neuropathy: evidence from genome-wide association studies and replication within multiple myeloma patients. BMC cancer, 2018. 18(1): p. 820.

Martin-Guerrero, I., et al., Variants in vincristine pharmacodynamic genes involved in neurotoxicity at induction phase in the therapy of pediatric acute lymphoblastic leukemia. The pharmacogenomics journal, 2019. 19(6): p. 564-569.

Komatsu, M., et al., Pharmacoethnicity in paclitaxel-induced sensory peripheral neuropathy. Clinical Cancer Research, 2015. 21(19): p. 4337-4346.
